# Microsporidian *Nosema bombycis* hijacks host vitellogenin and restructures ovariole cells for transovarial transmission

**DOI:** 10.1371/journal.ppat.1011859

**Published:** 2023-12-07

**Authors:** Chunxia Wang, Bin Yu, Xianzhi Meng, Dan Xia, Boyan Pei, Xiangyou Tang, Guizheng Zhang, Junhong Wei, Mengxian Long, Jie Chen, Jialing Bao, Chunfeng Li, Guoqing Pan, Zeyang Zhou, Tian Li

**Affiliations:** 1 State Key Laboratory of Resource Insects, Southwest University, Chongqing, People’s Republic of China; 2 Chongqing Key Laboratory of Microsporidia Infection and Control, Southwest University, Chongqing, People’s Republic of China; 3 Guangxi Institute of Sericulture Science, Nanning, People’s Republic of China; 4 College of Life Sciences, Chongqing Normal University, Chongqing, People’s Republic of China; University of Melbourne, AUSTRALIA

## Abstract

Microsporidia are a group of obligate intracellular parasites that infect almost all animals, causing serious human diseases and major economic losses to the farming industry. *Nosema bombycis* is a typical microsporidium that infects multiple lepidopteran insects via fecal-oral and transovarial transmission (TOT); however, the underlying TOT processes and mechanisms remain unknown. Here, we characterized the TOT process and identified key factors enabling *N*. *bombycis* to invade the ovariole and oocyte of silkworm *Bombyx mori*. We found that the parasites commenced with TOT at the early pupal stage when ovarioles penetrated the ovary wall and were exposed to the hemolymph. Subsequently, the parasites in hemolymph and hemolymph cells firstly infiltrated the ovariole sheath, from where they invaded the oocyte via two routes: (I) infecting follicular cells, thereby penetrating oocytes after proliferation, and (II) infecting nurse cells, thus entering oocytes following replication. In follicle and nurse cells, the parasites restructured and built large vacuoles to deliver themselves into the oocyte. In the whole process, the parasites were coated with *B*. *mori* vitellogenin (BmVg) on their surfaces. To investigate the BmVg effects on TOT, we suppressed its expression and found a dramatic decrease of pathogen load in both ovarioles and eggs, suggesting that BmVg plays a crucial role in the TOT. Thereby, we identified the BmVg domains and parasite spore wall proteins (SWPs) mediating the interaction, and demonstrated that the von Willebrand domain (VWD) interacted with SWP12, SWP26 and SWP30, and the unknown function domain (DUF1943) bound with the SWP30. When disrupting these interactions, we found significant reductions of the pathogen load in both ovarioles and eggs, suggesting that the interplays between BmVg and SWPs were vital for the TOT. In conclusion, our study has elucidated key aspects about the microsporidian TOT and revealed the key factors for understanding the molecular mechanisms underlying this transmission.

## Author summary

In addition to horizontal transmission, certain species of microsporidia possess the capability of vertically transmitting to host offspring by invading parental oocytes, a process known as transovarial transmission (TOT). However, the pathways and mechanisms through which these parasites penetrate the host barrier system and enter oocytes remain unclear. This study elucidates a comprehensive process in which the microsporidian *Nosema bombycis* infects various components of the silkworm *Bombyx mori* ovariole sheath, including follicular cells (FCs) and nurse cells (NCs), ultimately reaching their destination within the oocytes. Within FCs and NCs, *N*. *bombycis* undergoes restructuring and forms large vacuoles for delivering themselves into the oocyte. Furthermore, we have identified *B*. *mori* vitellogenin (BmVg) and specific spore wall proteins (SWP12, SWP26, SWP30) from *N*. *bombycis* as key factors involved in TOT. Additionally, our findings showed that specific domains within BmVg (VWD and DUF1943) are essential for binding with these spore wall proteins. Overall, this study represents pioneering work that illustrates both the process by which *N*. *bombycis* infects host oocytes and identifies crucial molecules that play vital roles in TOT mechanism. These findings provide novel perspectives on understanding pathogen-host interactions while advancing our knowledge of TOT mechanisms employed by pathogens.

## Introduction

Microsporidia are a diverse group of parasitic organisms that have a wide distribution and infect various animal species, including humans and economically important animals such as silkworms, bees, shrimp, crabs, and fish [1–4]. Since the discovery of the first microsporidium *Nosema bombycis* in 1857 from silkworm *Bombyx mori* [5], more than 220 genera and 1,700 species of microsporidia have been identified [6,7]. In addition to horizontal transmission, certain microsporidia like *N*. *bombycis*, *Nosema heliothidis* and *Nosema plodia* possess the ability to vertically transmit infections by invading parental ovaries and oocytes. This mode of transmission is known as transovarial transmission (TOT) [8–11]. The TOT of *N*. *bombycis* in silkworms poses a significant threat to sericulture [12–14]. Furthermore, TOT has also been observed in crop pests *Spodoptera litura* and *Helicoverpa armigera* infected with *N*. *bombycis* [15], suggesting its potential use as a biological insecticide for pest control. Additionally, TOT has been widely reported among viruses, bacteria, protists and endosymbiotic microorganisms [16–19], highlighting its common strategy for pathogen dissemination.

Studies have documented microsporidian infections in the reproductive tissues and eggs of hosts, thus establishing TOT in these parasites. However, the precise process and molecular mechanism underlying TOT remain elusive [17]. For instance, microsporidian TOT in dipteran insects, like *Amblyospora*, *Culicospora* and *Edhazardia*, was speculated to be achieved by directly injecting an infectious sporoplasm into the oocyte; however, the injection process has not been observed [20–24]. Similarly, the confirmation of TOT in *Antonospora locustae* infecting *Locusta migratoria* and *Nosema* sp. infecting *Malacosoma americanum* was solely based on observations of parasites in embryonic tissues [25,26]. More detailed insights have been gained regarding the TOT of microsporidian *N*. *heliothidis* and *N*. *plodia*, which proliferate within nurse cells (NCs) before being transferred to oocytes [8,9]. Furthermore, infection of oocytes from follicular cells (FCs) through spore germination has been described in *Gammarus dueben* infected by *Nosema granulosis* [17,27]. Nevertheless, a comprehensive understanding of the complete process involved in microsporidian TOT within host organisms is yet to be fully investigated.

The molecular mechanisms of TOT have been studied in several microorganisms, including geminiviruses, endosymbiotic bacterial spiroplasma, and *Wolbachia* [18,19,28]. These investigations have revealed a conserved TOT mechanism that involves the host vitellogenin (Vg), which plays a crucial role in mediating oocyte infection. For instance, spiroplasma, a natural endosymbiont of *Drosophila melanogaster*, reaches the host oocytes by traversing through the intercellular space surrounding ovarian FCs and is subsequently endocytosed into the oocytes within yolk granules during vitellogenic stages of oogenesis [29]. Similarly, *Wolbachia* exploits the host Vg transportation system to translocate from tropharium to developing oocytes of *Laodelphax striatellus* [30]. Furthermore, viruses can exploit this same transportation system to overcome barriers such as germarium and epithelial plug of ovary. Rice stripe tenuivirus (RSV) in small brown planthopper (*L*. *striatellus*) [31] and tomato yellow leaf curl virus (TYLCV) in whiteflies (*Bemisia tabaci*) directly utilize host Vg for achieving TOT [32]. During this process, RSV and TYLCV intrude into the host oocyte via Vg absorption channel on cell membrane while their surface protein pc3 mediates interaction with Vg.

The polytrophic lepidopteran ovary is composed of multiple ovarioles that develop within the ovarian capsule during the larval stage. Upon entering the pupal stage, the ovarioles rupture from the ovarian capsule and are exposed to hemolymph for nutrient absorption, initiating follicle vitellogenesis. Each ovariole consists of a terminal filament, germarium, vitellarium, and stalk. The elongated vitellarium contains linearly arranged ovarian follicles in successive stages of oogenesis (pre-vitellogenesis, vitellogenesis, and choriogenesis). During vitellogenesis, FCs develop channels (patency) for transporting nutrients such as Vg from hemolymph while oocytes differentiate into an oocyte-nurse cell complex [33–35]. Vitellogenesis is essential for egg development and embryonic growth in oviparous animals. In insects, vitellogenesis involves the synthesis of Vg in the fat body, its secretion into the hemolymph, transportation through follicular channels, and final uptake into maturing oocytes via endocytosis mediated by the vitellogenin receptor (VgR) on the cell membrane [34,36–38]. The follicular channels serve as a pathway for vertical transmission of pathogens and endosymbionts. For instance, when gap junctions between FCs and oocytes were inhibited or when there was a mutation in the VgR gene, ZAM retrovirus failed to invade oocytes in *Drosophila melanogaster* [39]. Similarly, knocking-down VgR expression disrupted the channel and disabled *Babesia* parasites from transferring across ticks ovarian tissues [40]. Furthermore, host embryos have been observed to accumulate microsporidia within yolk granules [26], suggesting that both follicular channels and Vg transportation play crucial roles in microsporidian vertical transmission. Recent studies have also shown that *N*. *bombycis* can infect both FCs and NCs of *S*. *litura* and *H*. *armigera* [15], indicating that microsporidia likely invade oocytes through multiple pathways. However, despite these findings, the process and molecular mechanism underlying microsporidian TOT has yet to be elucidated.

In this study, we investigated the process of *N*. *bombycis* TOT in silkworms and elucidated the pivotal role of *B*. *mori* vitellogenin (BmVg) in this process. This groundbreaking study not only provides a comprehensive understanding of how *N*. *bombycis* invades host oocytes but also identifies the key molecular players involved in TOT.

## Results

### *N*. *bombycis* invades oocytes after ovariole exposure to hemolymph at the pupal stage

**Fig 1:**
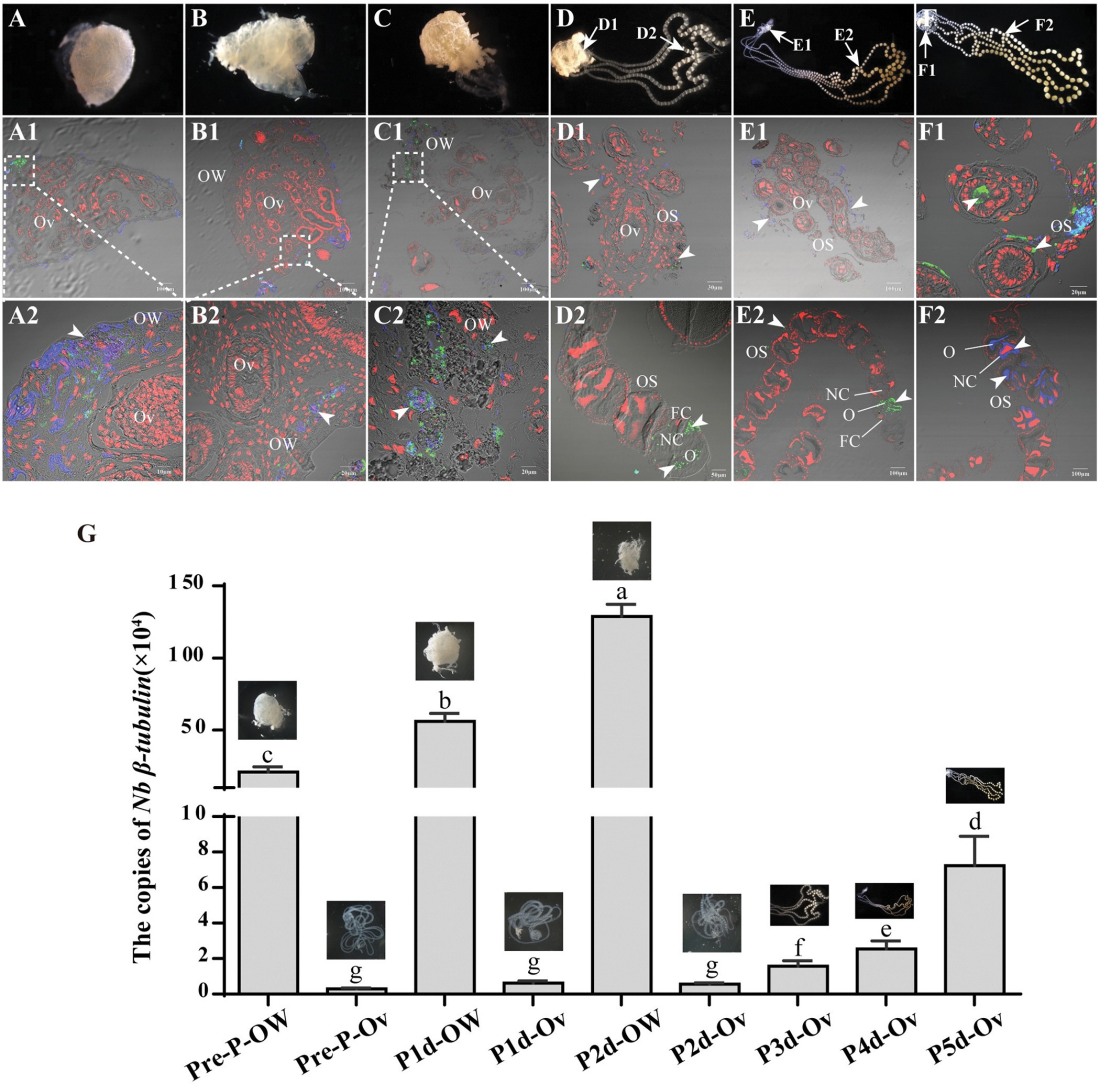
Silkworm ovaries and ovarioles infected by *N*. *bombycis*. Paraffin sections of the infected ovaries and ovarioles in the prepupal stage (A), and on the 1^st^ (B), 2^nd^ (C), 3^rd^ (D), 4^th^ (E) and 5^th^ (F) day of pupation were prepared. (A1-F1 and A2-F2) Immunofluorescence assay on the paraffin sections of the infected ovaries and ovarioles. Arrowhead shows *N*. *bombycis* in the ovarioles. *N*. *bombycis* spores were stained by FB28 (blue). Proliferative parasites were labeled with polyclonal antibodies against *N*. *bombycis* and conjugated to Alexa Fluor 488 (green). (G) Copies of *Nb-β-tubulin* were detected at six different stages in the ovarian wall and ovarioles. Pre-P, prepupal stage, P1d, 1^st^ day of pupation, P2d, 2^nd^ day of pupation, P3d, 3^rd^ day of pupation, P4d, 4^th^ day of pupation P5d, 5^th^ day of pupation. The data are described as the mean ± SD, and letters above the data columns indicate significant differences at the 0.05 level (independent-sample t test). All nuclei were stained with PI (red). OS, ovariole sheath; OW, ovarian wall; Ov, ovariole; FC, follicular cell; NC, nurse cell; O, oocyte.

To determine the precise timing of *N*. *bombycis* invasion into host oocytes, we meticulously observed morphological alterations in infected ovaries across various developmental stages. During the fifth instar, silkworm ovaries exhibited a distinct swelling and assumed a rounded shape, housing four ovarioles (Fig 1A). Following pupation, the ovarian capsule ruptured at its point of attachment to the oviduct, liberating the ovarioles into the abdominal cavity (Fig 1B and 1C). Subsequently, rapid development ensued within these ovarioles as follicles continued to proliferate and mature into eggs (Fig 1D–1F).

The pathogen loads in the ovarian wall and within the ovarioles were quantified using quantitative polymerase chain reaction (qPCR) with specific primers for *N*. *bombycis β-tubulin* during the pre-pupal stage and from the first to fifth day of pupation. The results revealed a continuous increase in pathogen load within the ovarian wall until two days prior to pupation, whereas noticeable infection of the ovariole was only detected after the third day of pupation (Fig 1G). Paraffin sections of the ovary demonstrated infection solely within the ovarian wall without any evidence of infection within the ovariole before the second day of pupation (Fig 1A1–1C2). Following three days of pupation, infection was observed within the ovarioles, with parasites present in nurse cells and oocytes (Fig 1D1–1F2). These observations indicate that *N*. *bombycis* is unable to invade the ovariole, until which breaches through the ovarian wall and becomes exposed to hemolymph.

### *N*. *bombycis* TOT begins with invading ovariole sheath

**Fig 2:**
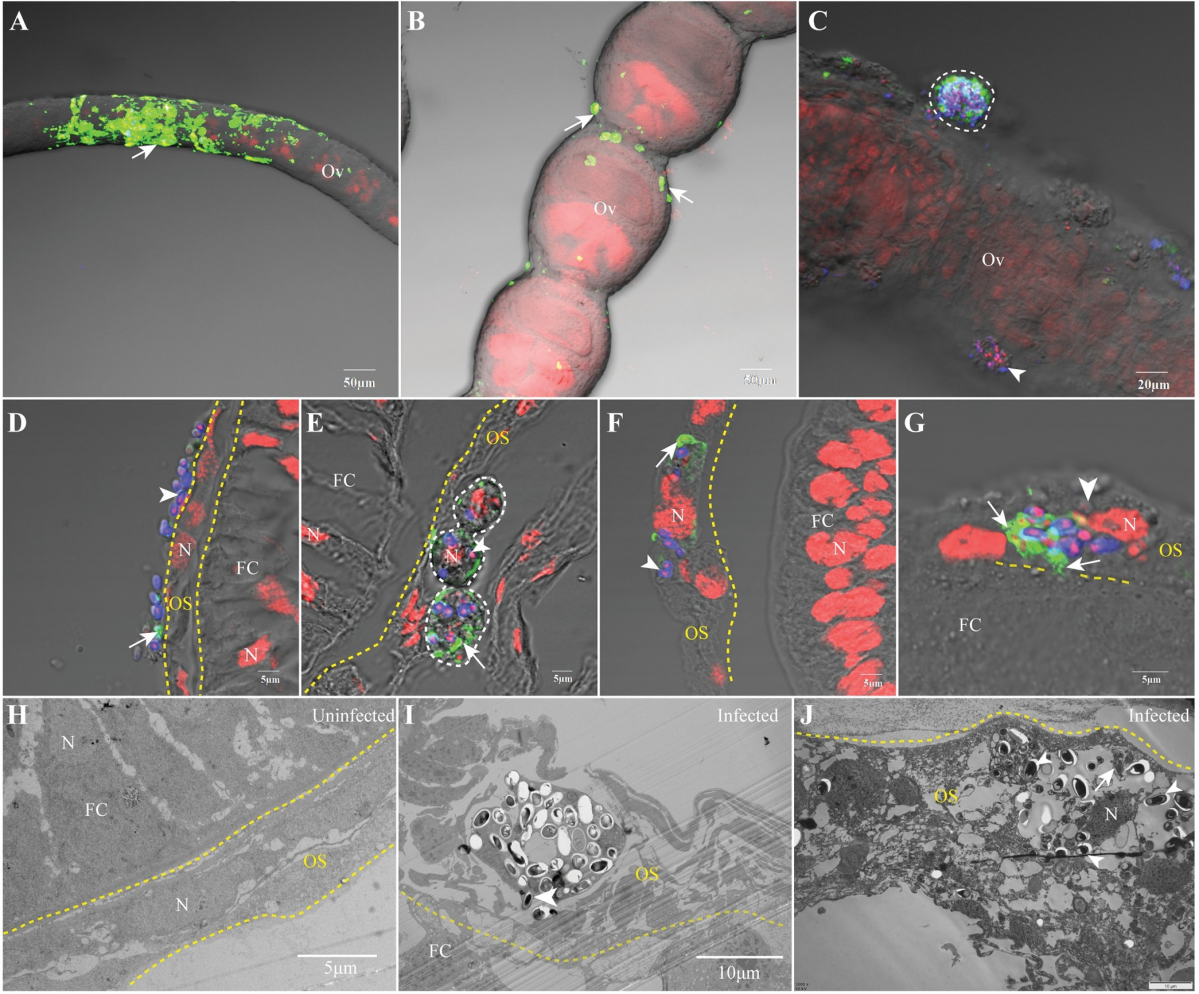
The infected silkworm ovarioles on the fifth day of pupation. The ovarioles infected by *N*. *bombycis* were analysed by IFA and TEM. (A-C) Infected ovarioles in previtellogenesis (A) and vitellogenesis stage (B). (D-F) IFA observations showed the adhesion of *N*. *bombycis* to ovariole sheath. (G) IFA demonstrated the parasite proliferation in the ovariole sheath. (H) TEM observation of the uninfected ovariole sheath. (I, J) TEM demonstrated the parasite infection and proliferation in the ovariole sheath. The yellow dashed lines indicate the boundaries of ovariole sheath, the white dashed lines indicate the infected hemolymph cells. Arrowhead, mature spores; arrow, parasites in proliferation; spores were stained using FB28 (blue); proliferative parasites was labeled with anti-*N*. *bombycis* polyclonal antibody conjugated with Alexa Fluor 488 (green); nuclei were stained using PI (red). N, nucleus; Ov, ovariole; OS, ovariole sheath; FC, follicular cell; O, oocyte; NC, nurse cell.

To elucidate the process by which *N*. *bombycis* invades oocytes from the hemolymph, we initially examined the overall infection of the ovarioles. Our immunofluorescence assay (IFA) results showed a severe infection of the ovariole sheath during both pre-vitellogenesis (Fig 2A and S1 Movie) and vitellogenesis stages (Fig 2B and S2 Movie). Adherence of parasites and infected hemolymph cells to the surface of the ovariole was observed (Fig 2C). Furthermore, we observed infection within FCs, NCs, and oocytes inside the ovarioles at both stages (S3 and S4 Movie). Additionally, infected hemolymph cells as well as free parasite spores in the hemolymph adhered to and infected the cells comprising the ovariole sheath (Figs 2D–2G and S1). The structure of these infected cells within the ovariole sheath exhibited severe destruction compared to uninfected cells (Fig 2H–2J). Following invasion of these ovariole sheath cells by parasites, massive replication occurred within them (Fig 2J), potentially leading to subsequent invasion into FCs from within the ovariole sheath itself (Fig 2G).

### *N*. *bombycis* invades and proliferates in FCs and NCs

**Fig 3:**
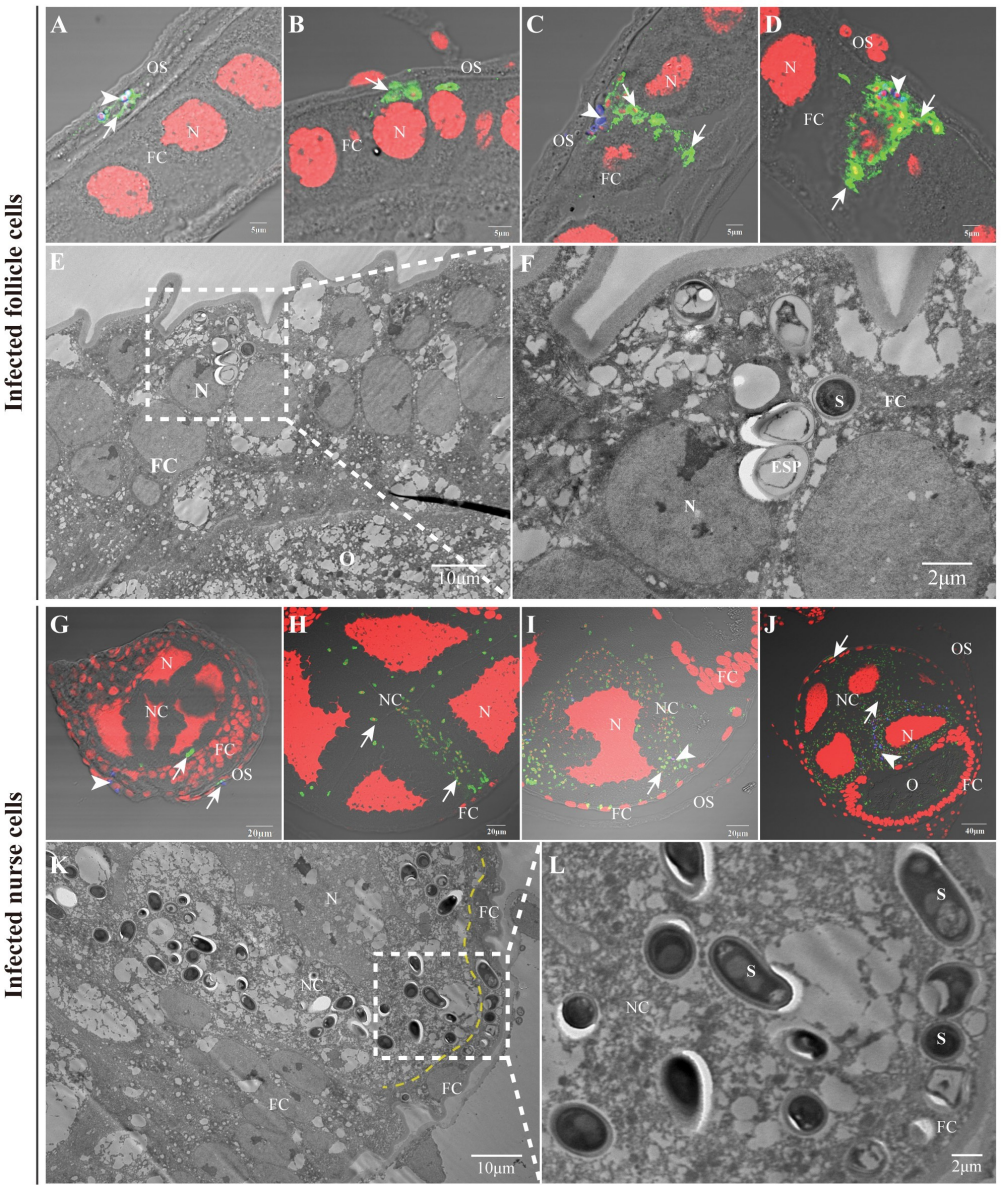
The infection of follicle and nurse cells by *N*. *bombycis*. (A-D) IFA showed that *N*. *bombycis* was infecting FC from ovariole sheath (A, B) and proliferated in FC (C, D). (E-F) TEM demonstrated the infection of FC by *N*. *bombycis*. (G-J) IFA displayed that *N*. *bombycis* infected NC from FC (G) and replicated in the NC (H-J). (K-L) TEM analysis of NCs infected by *N*. *bombycis*. The yellow dashed line indicates the boundary of FC and NC. Arrowhead, mature spores; arrow, proliferative *N*. *bombycis*; spores were stained using FB28 (blue); proliferative *N*. *bombycis* was labeled with anti-*N*. *bombycis* polyclonal antibody conjugated to Alexa Fluor 488 (green); Nucleus was stained using PI (red). N, nucleus; S, spore; ESP, empty spore shell; OS, ovariole sheath; FC, follicular cell; O, oocyte; NC, nurse cell.

In polytrophic meroistic ovarioles, each follicle consists of one oocyte and seven NCs surrounded by follicular epithelium. The oocyte and interconnected NCs are enclosed by a layer of FCs. To reach the oocyte, *N*. *bombycis* must traverse through the FCs and NCs. Following proliferation in the ovariole sheath, the parasites invade the FCs and undergo multiplication (Fig 3A–3F). We observed that the infection also spreads to the NCs located between the FCs and oocytes. Once invaded, the parasite continuously replicates within the NCs, leading to infection of neighboring cells (Fig 3G–3L). Eventually, adjacent cells become occupied by parasites (Fig 3J). We observed parasites at various stages and forms in both FCs and NCs (S2A–S2D Fig), including empty-shell spores (Figs 3F and S2D), suggesting that *N*. *bombycis* may employ polar tube ejection and schizogenic proliferation as its method of infection.

### *N*. *bombycis* invades oocytes from the FCs and NCs

**Fig 4:**
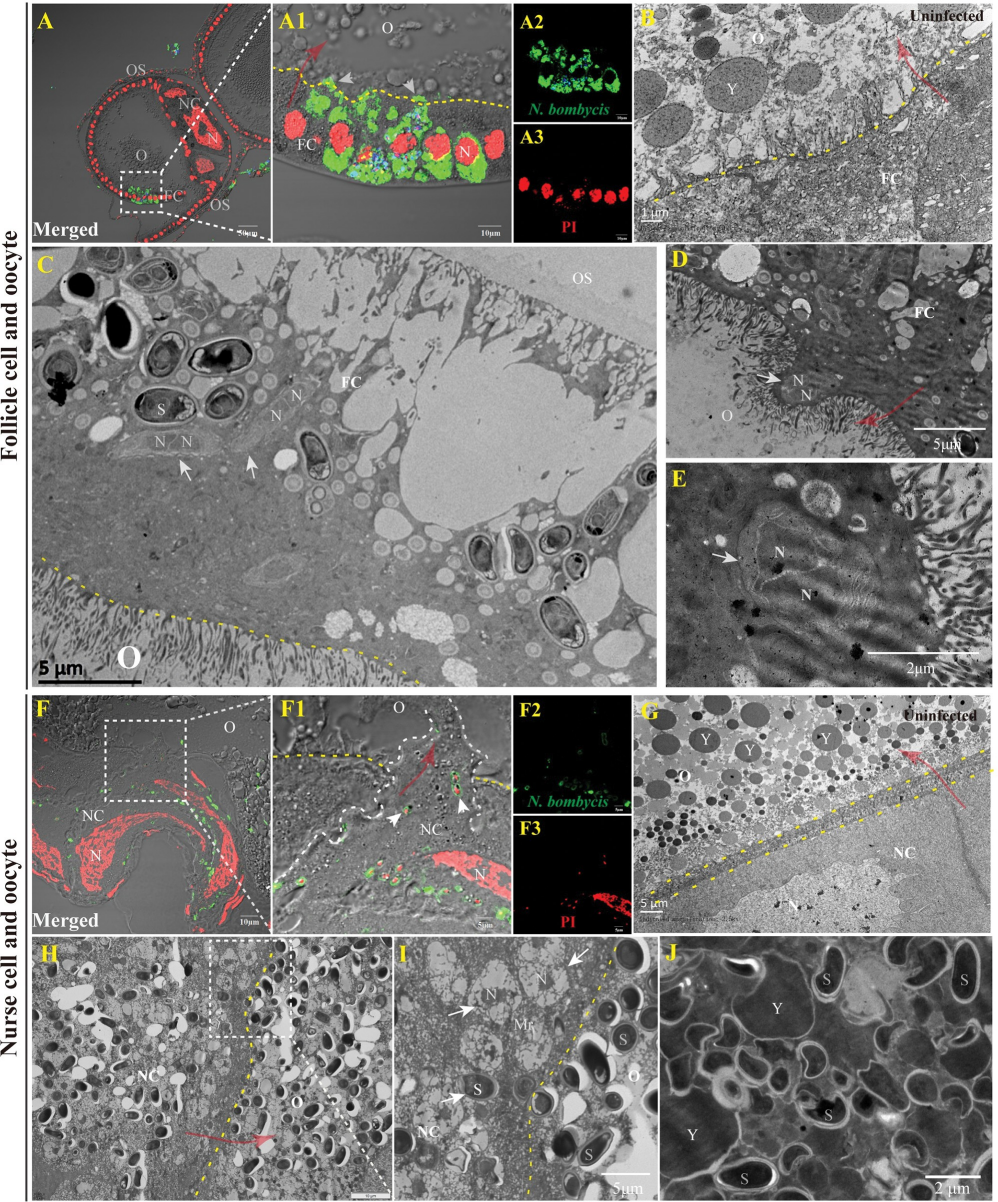
*N*. *bombycis* infected oocyte from the follicle and nurse cells. (A) IFA demonstrated the infection of oocyte by *N*. *bombycis* via the FC. (B) Observation of the uninfected FC and oocyte by TEM. (C-E) TEM showed the infection of oocyte by *N*. *bombycis* via the FC. (F) IFA illustrated the infection of oocyte by *N*. *bombycis* from the NC. (G) Observation of the uninfected NC and oocyte by TEM. (H, I) TEM displayed the infection of oocyte by *N*. *bombycis* from the NC. (J) *N*. *bombycis* in oocyte locates among the yolk granules. The arrow indicates the *N*. *bombycis*; spores were stained using FB28 (blue); the yellow dashed lines indicate the boundaries of cells; the red arrows indicate the direction in which the parasites enter the oocyte from FC or NC. *N*. *bombycis* in proliferation was labeled with anti-*N*. *bombycis* polyclonal antibody conjugated with Alexa Fluor 488 (green); Nuclei were stained by PI (red). N, Nucleus; S, spore; OS, ovariole sheath; FC, follicular cell; O, oocyte; NC, nurse cell; S, *N*. *bombycis* spores; Y, yolk granule.

The silkworm oocytes are connected to NCs and surrounded by FCs in order to absorb nutrients. Our observations have revealed two pathways through which *N*. *bombycis* reaches the oocyte. One pathway involves direct invasion of the oocyte from FCs (Figs 4A–4E, S2E and 2F). As depicted in Fig 4A, some parasites were observed passing through the FC-oocyte interface, where numerous vesicles extended into the oocyte (Fig 4C and 4D) for nutrient transport. Through TEM observation, we discovered large vesicles containing a parasite passing through this interface, almost entering the oocyte itself (Fig 4D). It was evident that the parasite within these restructured vesicles was not a mature spore but rather in a proliferative stage (Fig 4E). Alternatively, parasites within FCs invaded NCs and produced replicates that subsequently infected the oocyte by forming large vesicles capable of penetrating it (Figs 4F–4I, S2G and 2H). The parasites inside the oocytes continued to proliferate and were closely distributed among yolk granules (Figs 4J, and S2I, 2J).

### The ovariole infection is synchronous with BmVg expression

**Fig 5:**
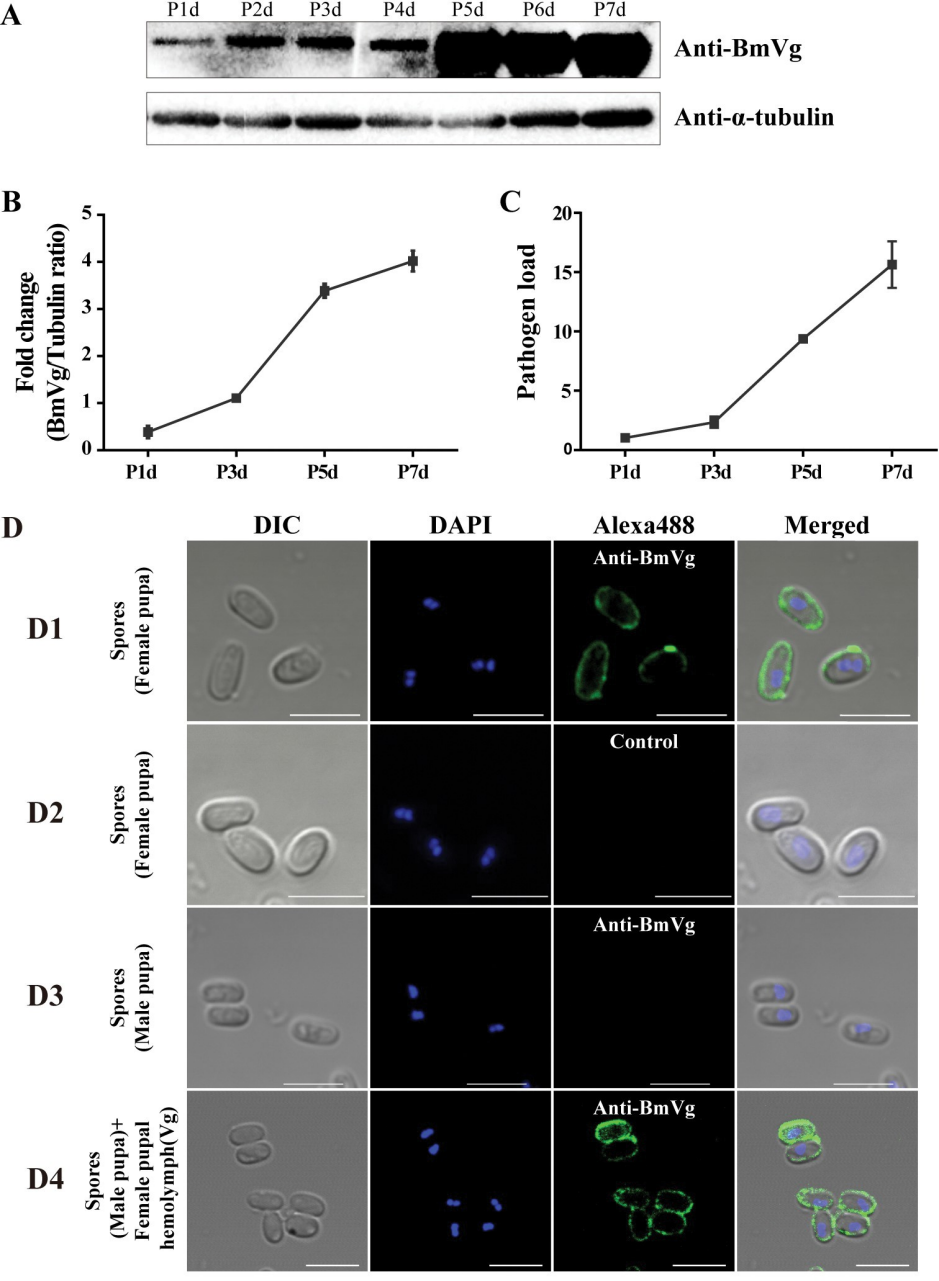
The ovariole infection was in sync with BmVg expression. (A) BmVg protein content in the ovarioles at different stage was determined by western blot using polyclonal antibody against BmVg (anti-BmVg) with *B*. *mori* α-tubulin (Bm-α-tubulin) as an internal reference. (B) Fold changes of BmVg content relative to Bm-α-tubulin in ovarioles. P1d, 1^st^ day of pupation, P3d, 3^rd^ day of pupation, P5d, 5^th^ day of pupation, P7d, 7^th^ day of pupation. (C) Fold changes of pathogen load indicated by the copy numbers of *N*. *bombycis β-tubulin* in ovarioles at different stages. Bars represent the average fold change per experiment ± SD. (D) IFA analysis of BmVg localization on the surface of *N*. *bombycis* spores. (D1) *N*. *bombycis* spores isolated from female pupae were labeled with the anti-BmVg. (D2) *N*. *bombycis* spores isolated from female pupae were incubated with negative serum as a control. (D3) *N*. *bombycis* spores isolated from male pupae were incubated with the anti-BmVg. (D4) *N*. *bombycis* spores isolated from male pupa were incubated with hemolymph of female pupa and labeled with the anti-BmVg. Nuclei were stained using DAPI (blue). The green fluorescence indicates the BmVg labeled by anti-BmVg conjugated with Alexa Fluor 488. Bars, 5 μm.

In oocytes, *N*. *bombycis* is closely associated with the yolk granules, which primarily consist of vitellin derived from the Vg protein [34,41], implying a potential correlation between TOT and Vg. Furthermore, BmVg exhibits specific and high expression levels in female silkworms after pupation. Hence, we examined BmVg expression during *N*. *bombycis* infection of the ovariole. Western blot analysis revealed a continuous increase in BmVg expression from the first to seventh day of pupation (Fig 5A and 5B). Concurrently, there was a significant rise in pathogen load within the ovarioles during this period (Figs 5C and 1G).

To confirm the potential involvement of BmVg in TOT, we investigated the interaction between BmVg and *N*. *bombycis* spores. Spores were isolated from the infected hemolymph of female and male pupae, and binding of BmVg to the spore surface was determined using an IFA. No presence of BmVg was observed on the surface of spores derived from male pupal hemolymph; however, it was specifically detected on the surface of spores obtained from female pupal hemolymph (Fig 5D). Furthermore, we confirmed this specific association between spores and BmVg through in vitro incubation experiments involving female hemolymph containing BmVg with spores purified from male silkworm larvae. As depicted in Fig 5D4, there was evident binding between spores and BmVg. These findings strongly suggest that BmVg plays a crucial role in facilitating *N*. *bombycis* TOT.

### *N*. *bombycis* is coated with BmVg in the whole process of TOT

**Fig 6:**
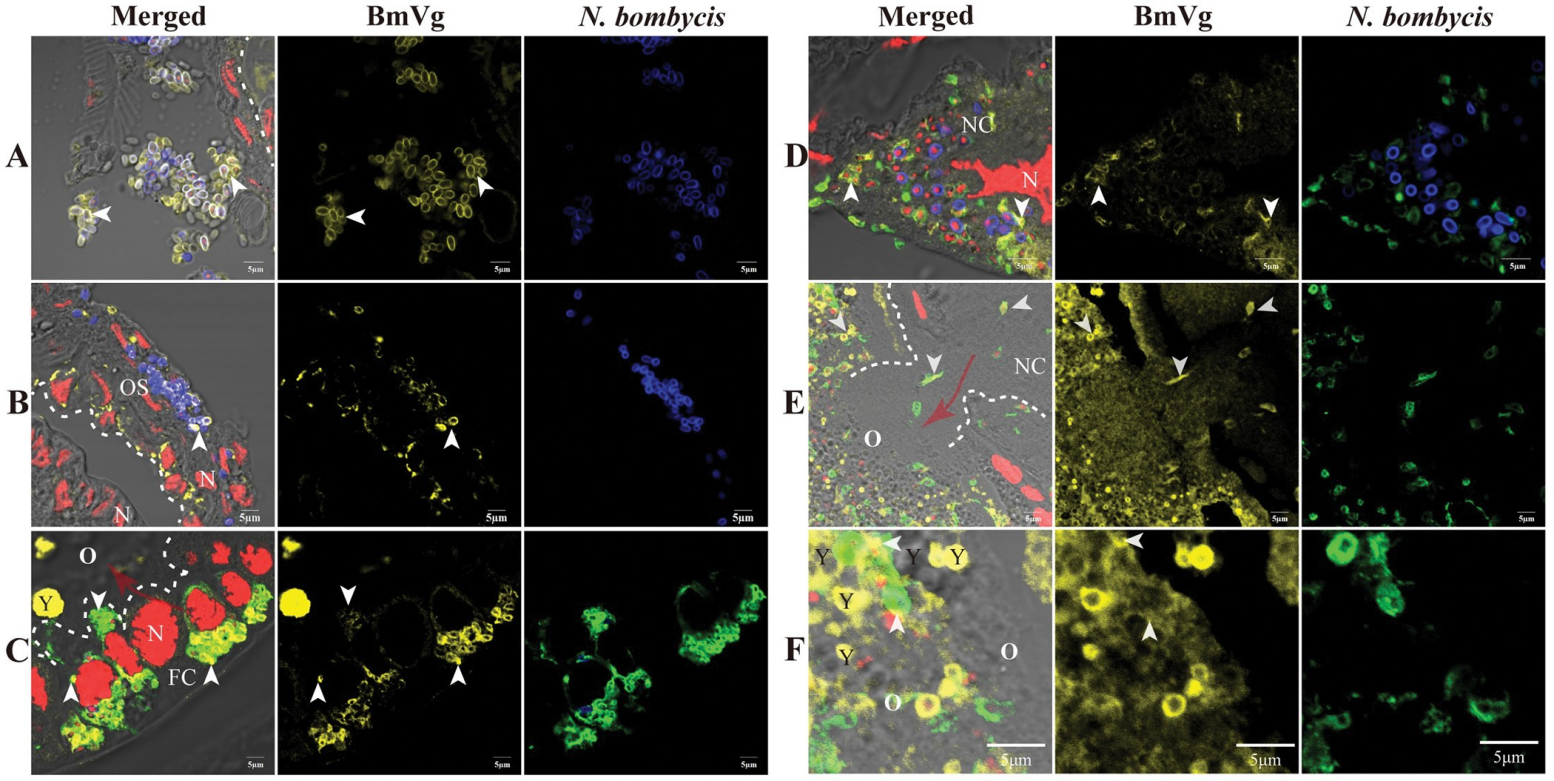
*N*. *bombycis* coated with BmVg in infected ovarioles. (A) *N*. *bombycis* in hemolymph coated with BmVg. (B) *N*. *bombycis* in ovariole sheath coated with BmVg. (C) *N*. *bombycis* coated with BmVg during infecting the oocyte by a restructured vacuole in FCs. (D) *N*. *bombycis* coated with BmVg in NCs. (E) *N*. *bombycis* coated with BmVg during infecting the oocyte by a restructured vacuole in NCs. (F) *N*. *bombycis* coated with BmVg surrounded by the yolk granules in oocyte. The white dashed lines indicate the boundaries of cells; the red arrows indicate the direction in which the parasites enter the oocyte from FC or NC. The arrowhead shows *N*. *bombycis* spores, which were stained with FB28 (blue); *N*. *bombycis* in proliferation was labelled by a rabbit anti-*N*. *bombycis* polyclonal antibody conjugated with Alexa Fluor 488 (green); the yellow fluorescence indicates the BmVg labeled by anti-BmVg conjugated with Alexa Fluor 647; nuclei were stained using PI (red). OS, ovariole sheath; FC, follicular cell; O, oocyte; NC, nurse cell; Y, yolk granule.

To verify the potential function of BmVg in the process of TOT, we examined the co-localization of BmVg with parasites. BmVg was found to be localized in various parts of uninfected ovarioles, including the ovariole sheaths, FCs, NCs, and oocytes (S3B1 Fig). Additionally, BmVg was observed on the surface of parasites during ovariole infection (Fig 6 and S4 Fig), specifically on mature spores adhering to both the surface (Fig 6A) and sheath (Fig 6B) of the ovarioles. Furthermore, we identified hemolymph cells containing parasites coated with BmVg within the ovariole sheaths (S4A and 4B Figs). In FCs (Figs 6C and S4C) and NCs (Fig 6D), clear evidence of BmVg coating on parasite surfaces was observed. Moreover, proliferative parasites invading oocytes from NCs were also coated with BmVg (Fig 6E). However, no adhesion between BmVgR and *N*. *bombycis* was detected (S3B4 Fig). Within the oocyte cytoplasmic region, *N*. *bombycis* was distributed among yolk granules while being coated with BmVg simultaneously (Figs 6F, S4D and 4E).

### RNAi of BmVg decreases the pathogen load in ovarioles and eggs

**Fig 7:**
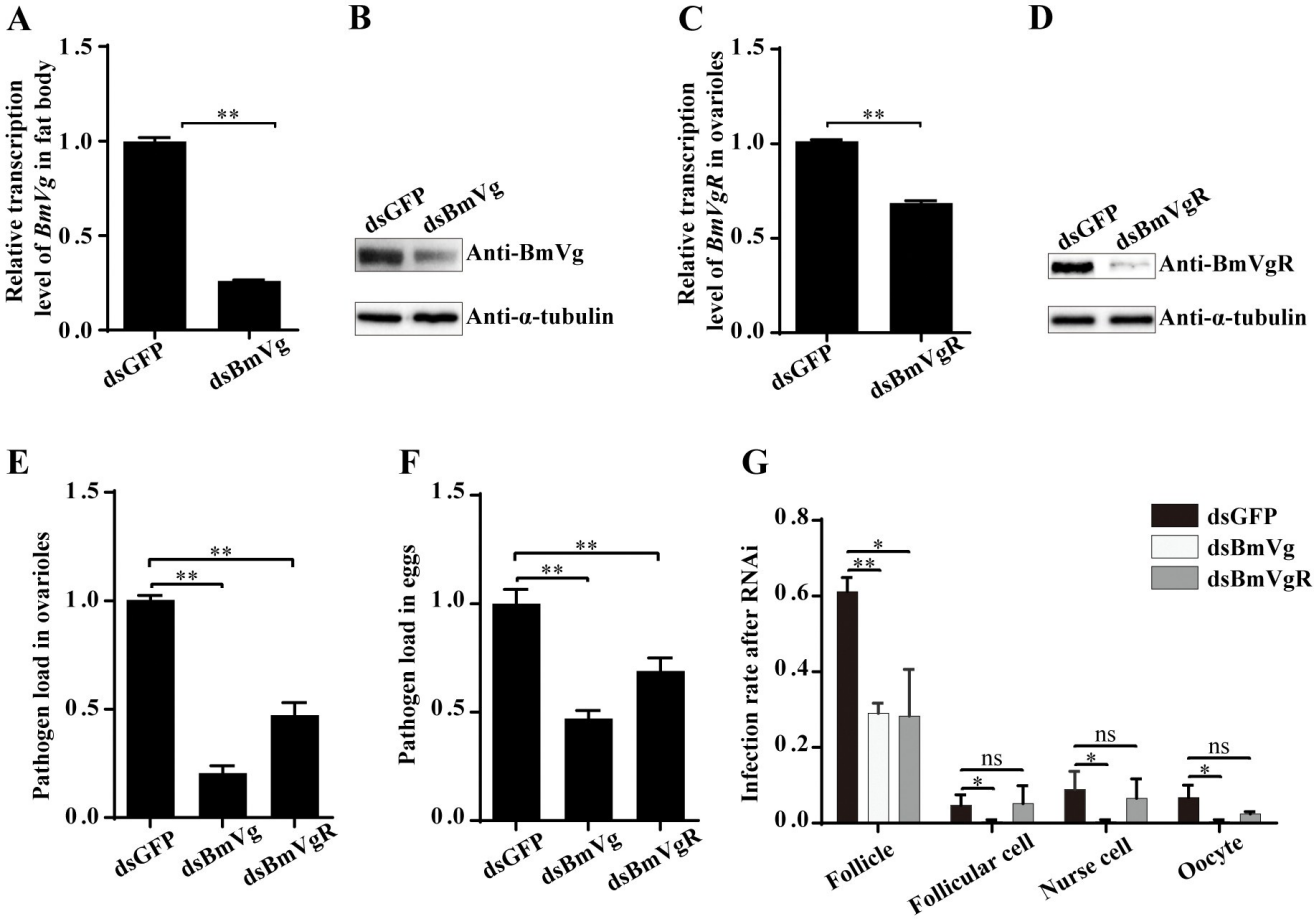
RNAi analysis on the role of BmVg in *N*. *bombycis* TOT. (A, B) RT-qPCR and western blot verification of the BmVg expression in silkworm fat body after RNAi. (C, D) RT-qPCR and western blot verification of the BmVgR expression in silkworm ovarioles after RNAi. (E, F) The pathogen load significantly reduced in ovarioles and eggs after RNAi of BmVg and BmVgR, respectively. (G) The infection rate significantly decreased in ovarioles, FCs, NCs and oocytes after knocking-down BmVG expression. The number of infected and total follicles were counted to calculate the infection rate of follicle. The infection rate of FC, NC and oocyte was represented by the number of follicles with an infection of FC, NC and oocyte to the total number of follicles, respectively. Bars represent the means ± SD from three independent experiments. *, *p* < 0.01; **, *p* < 0.01; ns, not significant (independent-sample t test).

Our findings suggest that BmVg likely plays a crucial role in the TOT of *N*. *bombycis*. To validate this hypothesis, we employed RNA interference (RNAi) to knock-down the expression of both BmVg and its receptor, BmVgR. As a result, there was a significant reduction of approximately 75% in BmVg expression in the fat body of fifth female pupae (Fig 7A and 7B). Additionally, the expression of BmVgR decreased by approximately 40% in the ovarioles (Fig 7C and 7D). The pathogen loads in the ovarioles were determined using qPCR with primers for *N*. *bombycis β-tubulin*, revealing a substantial decrease of around 80% and 50% upon RNAi targeting BmVg and BmVgR, respectively (Fig 7E). Similarly, there was also a significant reduction in pathogen loads within eggs (Fig 7F). However, it is worth noting that RNAi did not suppress infection within the fat bodies (S5A and S5B Fig). Furthermore, we confirmed the involvement of BmVg in *N*. *bombycis* TOT through IFA (S5C Fig), which results indicated that reduced infection rates within follicles after RNAi BmVg and BmVgR, respectively. After detecting the infection rate in FCs, NCs, and oocytes, we observed a significant decrease only following RNAi BmVg treatment, while no change was observed upon interfering with BmVgR (Fig 7G).

### The DUF1943 and VWD domains of BmVg mediate adhesion to the parasites

**Fig 8:**
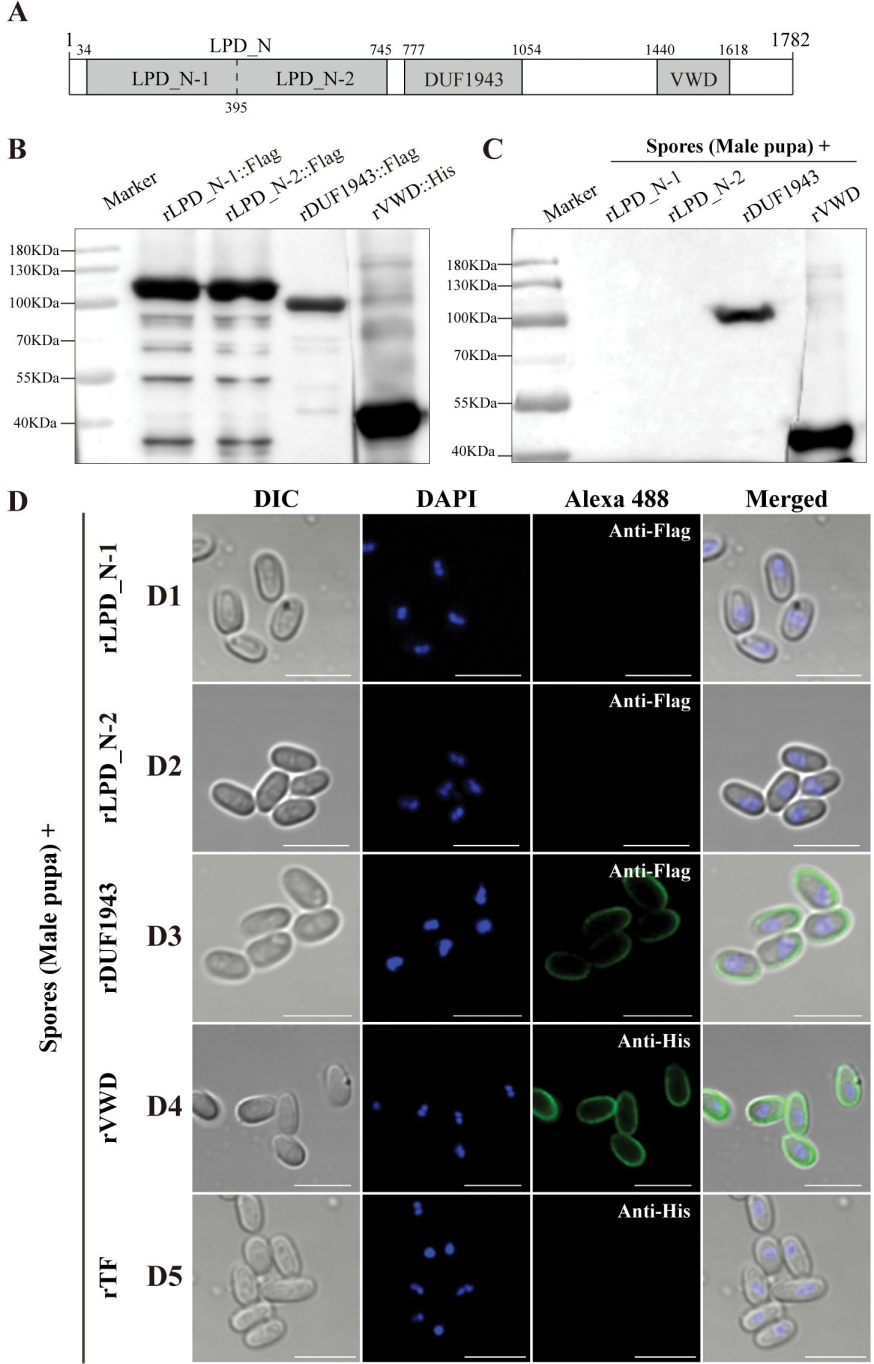
*N*. *bombycis* bound with BmVg domains VWD and DUF1943. (A) Predicted protein domains of BmVg. (B) Western blot analysis of the purified recombinant proteins of rLPD_N-1::Flag, rLPD_N-2::Flag, rDUF1943::Flag with an anti-Flag antibody and rVWD::His with an ant-His antibody. (C) Recombinant proteins binding to spores isolated from male pupae were detected using western blot analysis with anti-Flag and anti-His antibodies. (D) Immunofluorescence assay detection on the binding of recombinant proteins to spores isolated from male pupae. The rTF (solubility-enhancing tag proteins on the vector) was used as a control. Spore nuclei were stained using DAPI (blue). The green fluorescence indicated the antibody conjugated with Alexa Fluor 488. Bars, 5 μm.

The protein domains of BmVg were predicted to consist of a vitellogenin N domain (LPD_N), an unknown functional domain (DUF1943), and a von Willebrand domain (VWD) (Fig 8A). Recombinant proteins for the three domains, rLPD_N-1::Flag, rLPD_N-2::Flag, rDUF1943::Flag, and rVWD::His, were produced using the prokaryotic expression system and confirmed by western blot using Flag and His antibodies (Fig 8B). The recombinant proteins were incubated with male pupae-isolated *N*. *bombycis* spores to determine their binding affinity with BmVg through western blotting and IFA. As a result, the spores exhibited bindings with rDUF1943 and rVWD on their surface (Fig 8C and 8D), while no adhesion was observed between the spores and rLPD_N (Fig 8D1 and 8D2).

### BmVg binds with the spore wall proteins SWP12, SWP26 and SWP30

**Fig 9:**
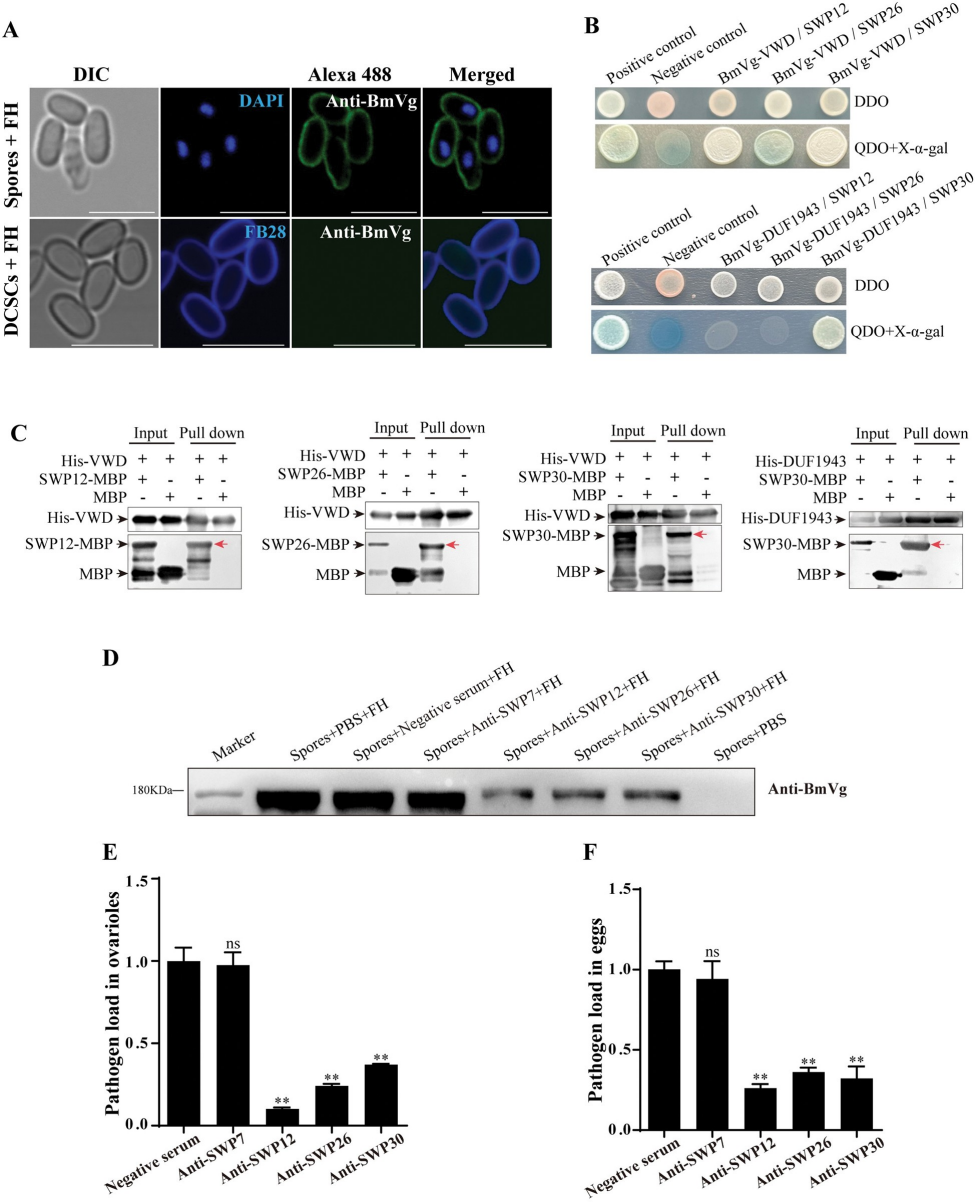
BmVg interacts with SWP12, SWP26 and SWP30. (A) Spores isolated from male pupae and deproteinized chitin spore coats (DCSCs) bound to female hemolymph (containing BmVg) labeled with anti-BmVg (Alexa488, green), spore nucleus was stained using DAPI (blue); DCSCs were labeled using FB28 (blue). Bars, 5 μm. (B) Yeast two-hybrid analysis of interactions among BmVg-VWD, BmVg-DUF1943 and SWP12, SWP26, SWP30. DDO, SD/-Leu/-Trp; QDO, SD/-Ade/-His/-Leu/-Trp.(C) An in vitro pull-down assay shows the interaction between VWD, DUF1943 and SWPs. (D) Western blot analysis on the binding of BmVg with the spores blocked by antibodies against SWP12, SWP26 and SWP30. FH, hemolymph of female pupae. (E) The pathogen load in ovarioles from pupae injected with SWP12, SWP26 and SWP30 was determined using qPCR with primers for *N*. *bombycis β-tubulin*. (F) The pathogen loads in eggs laid by pupae respectively injected with SWP12, SWP26, and SWP30 were determined using qPCR with primers for *N*. *bombycis β-tubulin*. Bars represent the means ± SD from three independent experiments. **, *p* < 0.01 (independent-sample t test).

The spore wall of microsporidia is composed of chitin and spore wall proteins (SWPs). Several SWPs from *N*. *bombycis* have been identified, including SWP5, SWP16, SWP32, SWP25, SWP26, and SWP30 [42,43]. To determine the molecules that BmVg binds to, we isolated deproteinized chitin spore coats (DCSCs) from mature spores as described in our previous study [44]. Subsequently, the DCSCs and complete spores were incubated with female pupae hemolymph, and then the BmVg binding was determined using IFA. Interestingly, only the complete spores exhibited BmVg signals on their surface while no such indications were observed on the DCSCs (Fig 9A). These findings suggest that parasites likely interact with BmVg through SWPs.

Subsequently, we employed the yeast two-hybrid system to elucidate the interactions between BmVg and SWPs (SWP5, SWP7, SWP9, SWP11, SWP12, SWP16, SWP25, SWP26, SWP30 and SWP32) [42,45,46]. Our findings revealed that the BmVg-VWD domain binds specifically with SWP12, SWP26 and SWP30; whereas the interaction of BmVg-DUF1943 domain is limited to only with SWP30 (Figs 9B and S6A, 6B). Furthermore, in vitro pull-down assays demonstrated that His-VWD successfully captured the SWP12-MBP, SWP26-MBP and SWP30-MBP; while His-DUF exclusively interacted with SWP30-MBP (Fig 9C). Additionally, IFA confirmed their colocalization on the spore wall by demonstrating co-localization of BmVg with SWP12, SWP26 and SWP30 (S6C Fig). To validate these interactions furtherly, we blocked spore surface using antibodies against the three SWPs followed by incubation with hemolymph isolated from female pupae for detection of BmVg binding through western blotting. Consequently, the coating of BmVg on spore surface was significantly reduced (Fig 9D). Moreover, to gain deeper insights into roles played by SWP12, SWP26 and SWP30 in *N*. *bombycis* TOT process, we injected corresponding antibodies into female pupae subsequently determined pathogen loads in ovarioles as well as eggs using qPCR, which resulted in the dramatic reductions of pathogen load in both ovarioles (Fig 9E) and eggs (Fig 9F).

### The proposed model for the TOT of *N*. *bombycis*

**Fig 10:**
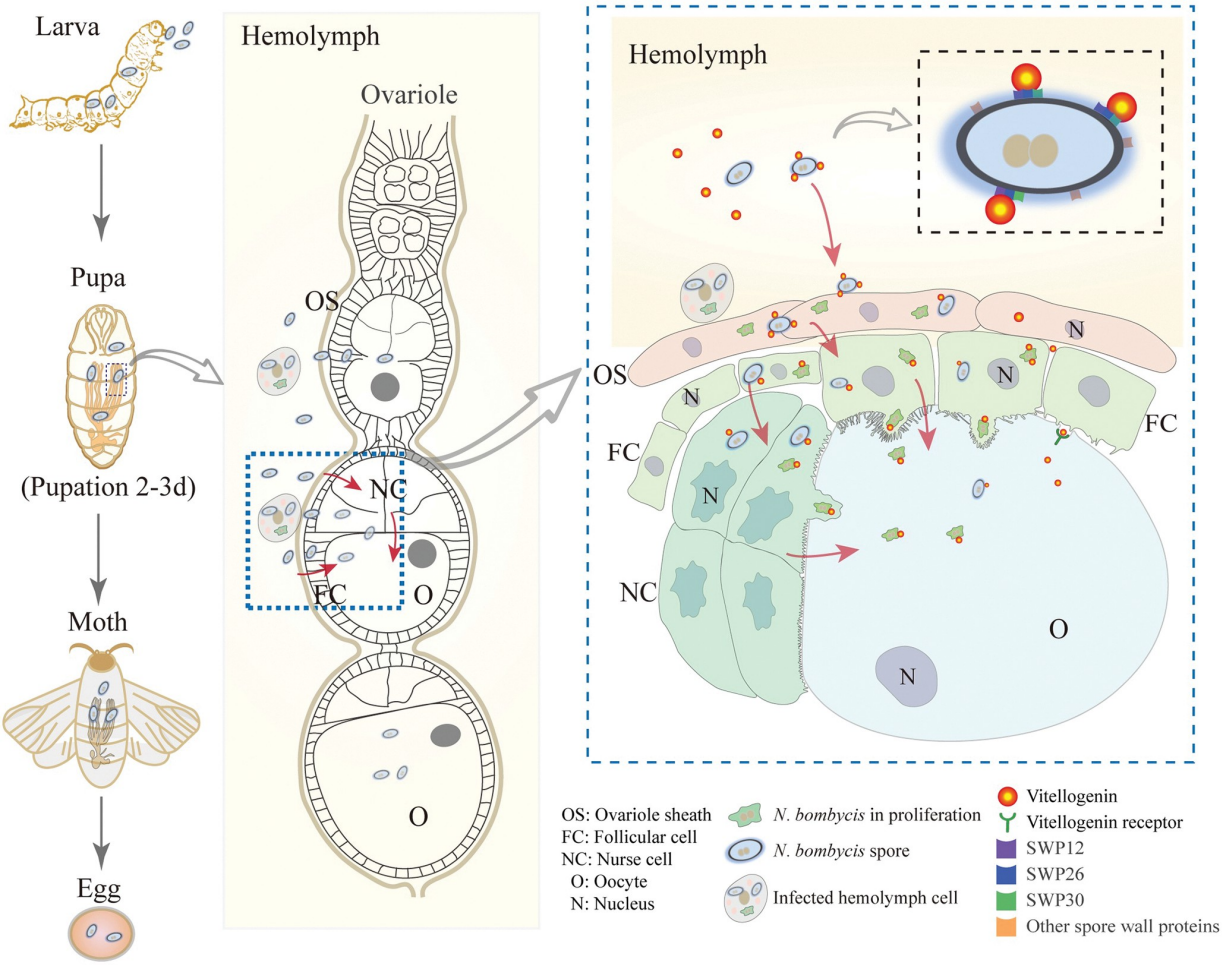
A model for elucidating the transovarial transmission of *N*. *bombycis*. During the vitellogenic stage, the ovarioles protrude from the ovary and are exposed to the hemolymph. *N*. *bombycis* in the hemolymph binds with the vitellogenin via spore wall proteins (SWPs), so that reaches and infects the ovariole sheath, follicular cells (FCs) and nurse cells (NCs). After replicating in the FCs and NCs, the parasites restructure a vesicle for delivering themselves into the oocyte to achieve the TOT.

By conducting this study, we propose a model for the TOT of *N*. *bombycis* (Fig 10). Two days after pupation, the ovarioles rupture from the ovary and are exposed to the hemolymph, where the parasites bind with BmVg in order to be transported and attach to the ovariole sheath (OS). Subsequently, the parasites invade OS cells and undergo replication before infecting FCs and proliferating once again. Within FCs, the parasites restructure a vacuole that allows direct entry into oocytes. Alternatively, they can infect NCs from FCs and multiply again before entering oocytes through restructured vacuoles. Throughout this process, it is likely that parasites bind with BmVg in order to move towards oocytes via nutrient transportation systems. In summary, TOT in *N*. *bombycis* is characterized by initiation during host vitellogenesis, hijacking Vg transportation using spore wall proteins, traversing an intracellular route to reach oocytes, and restructuring vacuoles within NCs and FCs for delivering large particles (parasites) into oocytes.

## Discussion

The TOT is a commonly employed strategy by certain pathogens [17–19]. Microsporidian TOT has been documented across multiple species [8,9,25,26,47–51]. These investigations primarily demonstrated the static localization and development of microsporidia within host germ cells. Only one study reported a partial progression from follicular cells to oocytes [47]. However, researchers have yet to investigate the complete process and molecular mechanism underlying microsporidian TOT.

Our study provides the first comprehensive characterization of the temporal sequence, complete pathways, process, and mediating proteins by which microsporidia invade the ovariole and oocyte. An intriguing finding is that the parasite induces severe restructuring of FCs and NCs. One notable structural change involves an increase in gap junction connections between FC-oocyte and NC-oocyte within infected follicles (Fig 4D and 4I) compared to uninfected ones (Fig 4G). These gaps are even replaced by large protrusions containing parasites that extend into the oocyte. These significant changes likely facilitate transportation of large particles like microsporidia into the oocyte (Fig 4D and 4E). This discovery reveals a novel mechanism through which larger parasites can invade a host’s oocytes.

More specifically, the infection of oocyte via NC and FC was commonly observed in polytrophic ovarioles, such as *N*. *bombycis*-infected *S*. *litura* and *H*. *armigera*, *Nosema plodiae*-infected *Plodia interpunctella*, *Nosema heliothidis*-infected corn earworms, and *Nosema pyrausta*-infected *Ostrinia nubilalis* [8,9,15,52]. Additionally, microsporidia also infected oocytes via FCs in telotrophic ovarioles [53]. This mode of infection differs from the TOT exhibited by certain endosymbiotic bacteria and plant viruses in telotrophic ovarioles [18,19,30,31,54,55], in which trophic cords and ovariole pedicel serve as channels for oocyte infection. The determinants of TOT routes remain unclear; however, particle size is likely a crucial factor influencing the transmission of these microorganisms.

To reach the host ovariole and oocyte, the transportation of pathogen particles is another crucial factor. During the vitellogenesis stage, abundant nutrients are synthesized in the fat body and secreted into the hemolymph to be transported to the ovariole. Subsequently, they are transferred to the oocyte surface through FC channels and finally endocytosed by the oocyte [31,34,36,37,56]. This conserved transportation system has been extensively exploited by various pathogens and endosymbiotic microorganisms for vertical transmission. For example, plant viruses like RSV, RGDV, and TYLCV can specifically bind with host Vg to invade the oocyte [19,31,32,56,57]. Endosymbiotic bacteria such as *Spiroplasma poulsonii* and Wolbachia also utilize Vg/VgR-mediated endocytosis for vertical transmission [29,30]. Therefore, it is likely that different microorganisms hijack host Vg for vertical transmission due to convergent evolution. As shown in S7 Fig, Vg exhibits high conservation among insects. Our study further demonstrates that *N*. *bombycis* directly interacts with Vg during vertical transmission not only in *B*. *mori* but also in *S*. *litura* and *H*. *armigera* (S8 Fig). These findings suggest that evolutionary conservation and functional significance of Vg potentially facilitate its exploitation by microorganisms for vertical transmission purposes. Apart from Vg protein itself, other nutrient proteins required by the oocyte such as apolipoprotein, storage protein, transferrin, and 30K protein are also transported via hemolymph [58–62], indicating potential involvement of additional factors in the TOT of *N*. *bombycis*.

The functions of Vg extend beyond that of an energy reserve for embryonic development. For instance, Vg is comprised of three protein domains: LPD-N, DUF1943, and VWD [36,63]. LPD-N is essential for recognition by the receptor VgR [64,65]. Additionally, the DUF1943 and VWD domains not only contribute to yolk formation in oocytes but also participate in pathogen recognition [66–68]. Furthermore, both DUF1943 and VWD from *Patinopecten yessoensis* Vg and zebrafish Vg2 have demonstrated binding capabilities to lipopolysaccharides and lipoteichoic acid found on bacterial cell walls [67,69]. In *L*. *striatellus*, RSV specifically interacts with the VWD and DUF1943 domains to overcome barriers and enter host oocytes [31,32,56]. Similarly, *N*. *bombycis* can also bind with DUF1943 and VWB domains, suggesting a convergent evolution in different TOT strategies. Moreover, the parasites attach themselves to host Vg using specific surface proteins. This indicates that Vg possesses high-affinity broad-spectrum properties, which have been exploited in all reported TOTs.

The uptake of Vg by the oocyte relies on VgR-mediated endocytosis, a process that has been exploited by viruses, bacteria, and fungi for transmission [19,29,31,32,39,40]. However, it appears that *N*. *bombycis* TOT is independent of this endocytosis, as evidenced by the RNAi of BmVgR (Fig 7G). This could be attributed to the fact that VgR-mediated endocytosis is primarily designed for small particles and may not be suitable for larger ones like microsporidia. Therefore, *N*. *bombycis* likely employs a novel strategy involving remodeling a large vacuole extending into the oocyte (Fig 4D). This alternative mechanism might rely on BmVg since the infection rate of oocytes decreased after RNAi treatment (Fig 7G). However, the mechanism by which the parasite utilizes BmVg during oocyte infection remains unclear. Additionally, it is intriguing to explore how BmVg contributes to the infection of NC and FC (Fig 7G). One speculation is that the parasite seizes control of the host nutrient transportation system by binding with BmVg in order to reach these cells.

## Materials and methods

### Ethics statement

The approval for animal experiments was obtained from The Laboratory Animal Welfare and Ethics Committee of The Third Medical University, Chongqing, China. The agreement number is SYXK(渝)2017-0019.

### Preparation of *N*. *bombycis* spores

*N*. *bombycis* isolate CQ1 was isolated from infected silkworms in Chongqing, China, and preserved at the China Veterinary Culture Collection Center (No. 102059). Spore extraction and purification were optimized as described previously [70]. Infected pupal tissue was homogenized in sterilized water and filtered using thick cotton in a 10 mL centrifuge tube. The remaining liquid was centrifuged at 3,000 rpm for 5 min at 4°C, and the pellet was washed three times and resuspended in distilled water. The spores were purified by centrifugation at 10,000 × *g* for 20 min in 75% Percoll (17089101, Cytiva, USA). The purified spores were washed three times with sterilized water and then suspended in 1.0 mL of water, and stored at 4°C for use.

### Insect rearing and parasite infecting

Silkworms (*Bombyx mori* Dazao and Guican No. 5) were obtained from the Silkworm Gene Resources Center of Southwest University, Chongqing, China. Silkworm larvae were reared on fresh mulberry leaves at 28°C, 75% relative humidity, and 12:12 h light:dark photoperiod in a growth chamber. Freshly exuviated fourth instar silkworms were individually orally inoculated with *N*. *bombycis* at a concentration of 1 × 10^4^ spores/larva. Normal feeding was subsequently performed during silkworm pupation, and infected ovaries and the ovarioles were removed at different stages.

The larvae of *S*. *litura* and *H*. *armigera* were purchased from Jiyuan Baiyun Industrial Co., Ltd. (Henan, China). The larvae were reared with a fresh artificial diet at 26°C, 70% relative humidity, and 12 h light and dark. On the second day of the third instar, the larvae were fed 2 × 10^3^ spores of *N*. *bombycis* per larva. Adult insects were reared in a 10% sucrose solution to supply energy. All infected tissue samples were treated and experiments performed in the same manner as for silkworms.

### Paraffin section of ovary and ovariole

Infected ovaries and ovarioles on the pre-pupa from the first to fifth day after pupation were prepared for paraffin sectioning. The samples were fixed with 4% paraformaldehyde for 12 h at 4°C, dehydrated with a gradient series of ethanol (70%, 80%, and 90%) for 1 h each, and dehydrated with 95% and 100% ethanol twice for 30 min. After gradient dehydration, the slides were placed in an equivalent mixture of ethanol and xylene for 5 min, followed by placement in complete xylene for 3 min, and then embedded in paraffin. The samples were sectioned into 5 μm slices and placed on glass slides. After deparaffinization and hydration, the slices were incubated with Citrate Antigen Retrieval Solution (MM1603, Beyotime, China) and stored at 98–100°C for 20 min, blocked in phosphate-buffered saline (PBS) containing 10% goat serum and 5% bovine serum albumin for 1 h at 37°C, and washed three times with PBS. The samples were used for subsequent antibody labeling experiments.

### Microscopic observation

Infected ovaries and ovarioles at different stages were collected and observed using the Olympus SZX16 microscope (Olympus, Tokyo, Japan) with a 1× objective lens and a 10× eyepiece and photographed using the Olympus cell Sens Standard 1.18.

### Transmission electron microscopy (TEM)

The ovarioles (as described above) of the silkworms infected with *N*. *bombycis* were fixed in 2.5% glutaraldehyde immediately after excision. The fixed samples were washed in 0.1 M PBS (pH 7.2) four times (15 min each) and postfixed in 1% osmium tetroxide for 2 h, followed by four additional washes (15 min each) in 0.1 M PBS. Thereafter, the samples were dehydrated using an ascending ethanol series and 100% acetone, infiltrated with gradient Epon812 (SPI, USA) resin, sequentially embedded in absolute resin, and polymerized at 70°C for 48 h. A Leica EM UC7 ultramicrotome (Leica, Germany) was used to acquire ultrathin sections, which were placed on nickel grids and stained with 3% uranyl acetate, followed by lead citrate. The stained grids were rinsed six times with ddH_2_O, dried, and photographed using a JEM-1400 Plus transmission electron microscope (JEOL, Japan) at an acceleration voltage of 80 kV.

### Preparation of polyclonal antibody

All gene sequences were obtained from NCBI GenBank (https://www.ncbi.nlm.nih.gov/genbank). The domains were predicted using SMART. The coding regions of *B*. *mori* vitellogenin (XM_038020969) and *B*. *mori* vitellogenin receptor (HM172611.1) antigenic epitope-rich regions were PCR-amplified from *B*. *mori* cDNA using PrimeSTAR Max DNA Polymerase (R045B, TaKaRa, Japan) with specific primers (S1 Table) and cloned into the vector pET-30a (+) for mouse polyclonal antibody preparation. *S*. *litura* vitellogenin VWD domain and *H*. *armigera* vitellogenin VWD domain gene sequences were synthesized by Sangon Biotech (Shanghai, China) and cloned into the vector pET-30a (+) for mouse polyclonal antibody preparation. *SWP12* (EF683112), *SWP26* (EU677842), and *SWP30* (EF683101) of *N*. *bombycis* were amplified using genomic DNA of *N*. *bombycis* spores via PCR using PrimeSTAR Max DNA Polymerase with specific primers (S1 Table) and cloned into the vector pET-30a (+) for rabbit polyclonal antibody preparation. All recombinant plasmids were transformed into *E*. *coli* Rosetta (CD801-02, TransGen Biotech, China) and cultured in Luria-Bertani medium at 37°C to an OD 600 of 0.6. Recombinant protein expression was induced by adding 0.1 Mm isopropyl β-d-thiogalactopyranoside for 20 h at 16°C. Proteins were purified using a Ni^2+^-nitrilotriacetic acid column (30210, QIAGEN, Germany) according to the manufacturer’s instructions. Antisera were generated from BALB/c mice by immunization with the recombinant protein (100 μg each time) and homogenized using Freund’s adjuvant (F5505, Sigma, USA). After four weekly immunizations, the antisera were collected and stored at −20°C until use. Control sera were collected from BALB/c mice prior to immunization (pre-immune serum). New Zealand White rabbits were used to generate antisera via immunization with the recombinant protein (1 mg each time) mixed with Freund’s complete adjuvant (F5881, Sigma) to form a stable emulsion for immunization. The rabbits were injected subcutaneously at four sites and boosted four times, once a week. The antisera were collected and stored at −20°C until use.

### Immunofluorescence assay (IFA)

#### Detection of *N*. *bombycis* infection in ovarioles

To better observe *N*. *bombycis* infection in ovarioles, we performed tissue transparency treatment and rabbit anti-*N*. *bombycis* polyclonal antibody [11] (1:200 dilution) labeling using the CytoVista Tissue and 3D Culture Clearing Kit (V11324; Invitrogen, Carlsbad, CA, USA) according to the manufacturer’s instructions. Z-axis depth photography was subsequently employed using an Olympus Biological Confocal Laser Scanning Microscope FV1200 (Olympus, Tokyo) at 200× magnification to clearly display the infection.

#### Detection of *N*. *bombycis* infection in paraffin sections of ovarioles

To observe the distribution of *N*. *bombycis* in ovarioles, the slices were incubated with rabbit anti-*N*. *bombycis* polyclonal antibody (1:200) at 37°C for 90 mins, washed three times with PBST (0.01 M PBS + 0.05% Tween 20) each for 5 min, followed by incubation with goat anti-rabbit secondary antibody labeled with Alexa Flour 488 (A32731, Invitrogen), fluorescent brightener 28 (FB 28) (ZY4404, China) for staining chitin, and propidium iodide (PI) (P3566, Invitrogen) for staining nuclei in a dark environment for 40 mins. The slides were washed three times with PBST, then suspended in Fluoromount Aqueous Mounting Medium (F4680, Sigma) cover glass.

#### Detecting the localization of BmVg on the spore surface

To observe the localization of BmVg within spores in infected silkworms, spores were isolated from the hemolymph of the pupae, washed, and fixed. They were subsequently blocked with tyrosine buffer and incubated with anti-BmVg (1:100 dilution) for 2 h at 37°C, followed by incubation with Alexa 488-labeled secondary antibody, and 4,6-diamidino-2-phenylindole (DAPI) (D9542, Sigma) for detecting nuclei.

#### Detecting the colocalization of BmVg with *N*. *bombycis*

To observe the colocalization of BmVg and *N*. *bombycis* in ovarioles, the sections were incubated with rabbit anti-*N*. *bombycis* polyclonal antibody (1:200), mouse anti-BmVg polyclonal antibody (1:200), or mouse anti-BmVgR polyclonal antibody (1:200) at 37°C for 90 min and washed three times with PBST each for 5 min, followed by incubation with goat anti-rabbit secondary antibody labeled with Alexa Flour 488, goat anti-mouse secondary antibody labeled with Alexa Flour 647 (A32728, Invitrogen) and washed three times with PBST for 5 min each. Finally, the sections were incubated with a mixed solution of FB 28 and PI to stain the chitin and nuclei, respectively.

The deproteinized chitin spore coats (DCSCs) of *N*. *bombycis* were extracted as previously described [44]. The DCSCs and complete spores were incubated with the hemolymph of female pupae for 2 h at 28°C, and then the BmVg binding was determined using IFA. BmVg was labeled with secondary antibodies coupled with Alexa Fluor 488, and the chitins and nuclei were stained with FB 28 and DAPI, respectively.

#### Detecting the colocalization of BmVg with SWP12, SWP26, and SWP30

To confirm these interactions between BmVg and SWP12, SWP26, and SWP30, we firstly incubated spores with female pupal hemolymph, and then detected the colocalization of BmVg with SWP12, SWP26, and SWP30 on the spore surface using IFA. BmVg was labeled with secondary antibodies coupled with Alexa Fluor 488. SWP12, SWP26, and SWP30 were stained using a secondary antibody coupled with Alexa Fluor 594 (A32740, Invitrogen), and the spore nuclei were stained with DAPI. Finally, the samples were observed and photographed using an Olympus Biological Confocal Laser Scanning Microscope FV1200 at 200× and 1000× for global and local observations, respectively.

### Protein extraction and western blot assay

The silkworm fat body and ovarioles were ground in liquid nitrogen, and the proteins were extracted using lysis buffer (20 mM Tris, pH 7.4, 0.15 M NaCl, 1 mM EDTA, 0.1% Triton-X, 0.1% sodium dodecyl sulfate). The samples were centrifuged at 13,000× *g* for 30 min, the supernatant collected, and the protein concentration quantified using the bicinchoninic acid protein assay (P0010S, Beyotime). For immunoblotting analysis, total proteins were separated using 8% sodium dodecyl-sulfate polyacrylamide gel electrophoresis (SDS-PAGE) and transferred to a polyvinylidene difluoride (PVDF) membrane (Roche, Switzerland). The membrane was incubated with anti-BmVg (1:500) for 1 h at 37°C. Thereafter, it was washed three times with Tris-buffered saline containing 0.05% Tween 20 (TBST), and incubated for 1 h at 37°C with HRP-linked anti-mouse IgG antibody (BL001A, Bioshap, Hefei, China). After three washes with TBST, the membrane was exposed to an ECL western blot detection kit (34580, Thermo Fisher Scientific) and imaged using an Azure Biosystems imaging system (C300, USA).

### Detection of pathogen load by qPCR

To determine the *N*. *bombycis* infection status of ovarioles and ovarian walls at different time points, genomic DNA was extracted from the samples using the EZNA Tissue DNA Kit (D3396-02, OMEGA, USA) according to the manufacturer’s instructions. The genomic DNA was used as the template, and the initial copy number of *N*. *bombycis β-tubulin* (EOB14994.2) [70] was calculated using qPCR with specific primers (S1 Table). 1.8 × 10^8^ copies of the recombinant pMD 19-T Vector plasmid (6013, TaKaRa) integrated with *N*. *bombycis β-tubulin* was used as the standard. qPCR was performed as following procedure, pre-denaturation at 95°C for 2 min, followed by 40 amplification cycles at 95°C for 10 s and 60°C for 20 s (LightCycle 96, Roche). The infection rate was calculated as the *N*. *bombycis β-tubulin* copy number ratio between the experimental and control groups.

### RNAi via injection of dsRNA

We then used RNAi strategy to determine the functional roles of BmVg and BmVgR during the *N*. *bombycis* infection in *B*. *mori*. Briefly, dsRNAs targeting 500 bp regions of *BmVg*, 692 bp regions of *BmVgR* or *green fluorescent protein* (*GFP)* genes were synthesized using the T7 RiboMAX Express RNAi System (P1700, Promega, USA) according to the manufacturer’s instructions. The specific primers used to generate the DNA templates are listed in S1 Table. The acquisition, injection dose, and injection process of double-stranded RNA (dsRNA) were performed as previously described [71] with minor modifications. Female silkworms on the first day of the wandering and the first day and third day of pupation were injected with 20 μg of ds*BmVg* or ds*BmVgR*. An equal volume of dsGFP was simultaneously injected as a control. Thereafter, the fat bodies and ovarioles were collected to quantify the transcripts and proteins expression of *BmVg* and *BmVgR*, respectively. The copy number of *N*. *bombycis β-tubulin* in the fifth day of pupation after injection was determined using qPCR with specific primers (S1 Table). The samples were prepared in the same manner as the paraffin section samples to detect pathogen distribution. Total RNA was extracted using the EZNA Total RNA Kit II (R6934-01, OMEGA) according to the manufacturer’s instructions. The cDNA was synthesized with 1 μg total RNA using the GoScript Reverse Transcription System Kit (A5003, Promega) after DNA digestion with DNase I. Relative BmVg and BmVgR mRNA levels were measured using qPCR with specific primers and *RPL3*-specific primers (S1 Table) as follows: a pre-denaturation of 95°C for 2 min, followed by 40 cycles at 95°C for 10 s, and 60°C for 20 s (LightCycle 96, Roche). The relative gene expression level was calculated using the 2^-△△Ct^ method [72]. The remaining adults and pupae were maintained at 26°C. After eclosion, dsRNA-injected *B*. *mori* moths laid eggs, and the quantity of *N*. *bombycis* was measured as the copy numbers of *N*. *bombycis β-tubulin* as mentioned above.

### Identification of BmVg domains binding with the parasites

To investigate whether different domains bind *N*. *bombycis* spores, recombinant proteins of different Vg domains: rLPD_N-1::Flag, rLPD_N-2::Flag, rDUF1943::Flag and rVWD::His were expressed and purified separately. Recombinant proteins were incubated with *N*. *bombycis* spores purified from male pupae for 2 h at 28°C. The mixtures were centrifuged for 5 min at 3,000 × *g*, and the supernatants were discarded. After centrifugation and washed five times with PBST, the spore-bound proteins were separated by SDS-PAGE gel electrophoresis and transferred to a PVDF membrane. Anti-Flag (F1804, Sigma) was used for western blot verification of purified recombinant rLPD_N-1::Flag, rLPD_N-2::Flag, and rDUF1943::Flag, Anti-His (AH367, Beyotime) was used for western blot verification of purified recombinant rVWD. The same sample was also used for the IFA. The operation was as previously described above.

### Yeast two-hybrid assay

BmVg-VWD, BmVg-DUF1943, and spore wall proteins (SWP5, SWP7, SWP9, SWP11, SWP12, SWP16, SWP25, SWP26, SWP30, and SWP32) were amplified and cloned into bait (pGBKT7) or prey (pGADT7) plasmids. The bait and prey plasmids were co-transformed with the yeast strain Y2HGold (MF2351, MKBio, China). The positive control pGBKT7-53/pGADT7-T and negative control pGBKT7-Lam/pGADT7-T were transformed in the same manner. The cultured strains were placed on an SD/-Ade/-His/-Leu/-Trp with X-α-gal (QDO) solid culture plate for screening.

### In vitro pull-down assay

The full-length coding sequences of SWP12, SWP26, and SWP30 were amplified and cloned into the pMAL-c5X vectors. Subsequently, they were introduced into Rosetta (DE3) chemically competent cells to express SWP12-MBP, SWP26-MBP, and SWP30-MBP proteins. Soluble MBP fusion proteins were extracted using an MBP Sep Dextrin Agarose Resin 6FF (20515ES25). His-VWD or His-DUF1943 protein was incubated with MBP and SWPs-MBP protein using the Dynabeads His-Tag Isolation & Pulldown kit (10103D; Invitrogen), following the manufacturer’s instructions. The interaction was detected by western blot analysis using an anti-MBP antibody.

### Antibody blocking analysis

To analyze the function of SWP12, SWP26 and SWP30, spores were incubated with the antibodies (anti-SWP7, anti-SWP12, anti-SWP26, and anti-SWP30) at 4°C for 12 h and were subsequently incubated with female hemolymph (BmVg) for 2 h at 4°C. The samples were washed three times with PBST. The amount of BmVg protein that adhered to the surface of *N*. *bombycis* spores was detected using western blotting.

### Injection of antibodies against SWPs

To explore the role of the interaction between SWPs and BmVg in *N*. *bombycis* TOT, we injected polyclonal antibodies against the SWPs (anti-SWP7, anti-SWP12, anti-SWP26, and anti-SWP30) into infected silkworms on the first day of the wandering, and the first and third day of pupation to block the interaction in the hemolymph. The samples were prepared in the same manner as the paraffin section samples to detect pathogen distribution. Thereafter, we conducted qPCR to detect the pathogen load of *N*. *bombycis* after extracting DNA as described above. After eclosion, antibody-injected *B*. *mori* moths laid eggs, where the quantity of *N*. *bombycis* was measured as the copy number of *N*. *bombycis β-tubulin* as previously mentioned.

## Supporting information

S1 Fig IFA and TEM observations show the infected ovariole sheath. IFA observations show the adhesion of *N*. *bombycis* to ovariole sheath (A, B) and proliferation (C). (D-E) TEM demonstrate the parasite proliferation in ovariole sheath. The yellow dashed lines indicate the boundaries of ovariole sheath; white dashed lines indicate the infected hemolymph cells; red dashed lines indicate lipid droplet in the cell. Arrowhead, mature spores; arrow, parasites in proliferation; spores were stained using FB28 (blue); proliferative parasites was labeled with anti-*N*. *bombycis* polyclonal antibody and conjugated with Alexa Fluor 488 (green); nuclei were stained using PI (red). N, nucleus; Ov, ovariole; OS, ovariole sheath; LD, lipid droplet; FC, follicular cell; O, oocyte; NC, nurse cell.
(TIF)

S2 Fig TEM demonstrated the infection of follicle and nurse cells by *N*. *bombycis*. (A, B) TEM demonstrated the infection of FC by *N*. *bombycis*. (C, D) TEM analysis of NCs infected by *N*. *bombycis*. (E, F) TEM showed the infection of oocyte by *N*. *bombycis* via the FC. (G, H) TEM showed the infection of oocyte by *N*. *bombycis* from the NC. (I, J) *N*. *bombycis* in the oocyte was distributed around the yolk granules. The yellow dashed lines indicate the boundaries of cells. The arrow indicates the *N*. *bombycis*; spores were stained using FB28 (blue); *N*. *bombycis* in proliferation was labeled with anti-*N*. *bombycis* polyclonal antibody conjugated with Alexa Fluor 488 (green); nuclei were stained by PI (red). N, nucleus; S, spore; ESP, empty spore shell; OS, ovariole sheath; FC, follicular cell; O, oocyte; NC, nurse cell; Mr, meront; Sp, sporont; Sb, sporoblast.
(TIF)

S3 Fig Detection of polyclonal antibodies prepared in this study. (A) Detection of polyclonal antibodies against BmVg and BmVgR using western blotting. (B) Subcellular localization of BmVg and BmVgR in ovarioles. (B1) Localization of BmVg in uninfected ovarioles. (B2) Negative serum was used as the control. (B3) Localization of VgR in uninfected ovarioles. (B4) Localization of *N*. *bombycis* spores and BmVgR in oocytes. (C) Detection of polyclonal antibodies that recognize the *N*. *bombycis* spore wall protein 12, spore wall protein 26, and spore wall protein 30 using western blotting. The arrowhead shows *N*. *bombycis*; *N*. *bombycis* spores were stained using FB28 (blue); proliferative *N*. *bombycis* were labelled with rabbit polyclonal antibody against *N*. *bombycis* (Alexa488, green); BmVg was detected using a mouse anti-BmVg polyclonal antibody (Alexa647, yellow); nuclei were stained using PI (red). OS, ovariole sheath; FC, follicular cell; O, oocyte; NC, nurse cell.
(TIF)

S4 Fig *N*. *bombycis* coated with BmVg in hemolymph and ovariole sheath. (A, B) *N*. *bombycis* coated with BmVg in the ovariole sheath cells, the white dashed lines indicate the infected hemolymph cells. (C) *N*. *bombycis* coated with BmVg during infecting the oocyte from FCs, the white dashed lines indicate the boundaries of FCs. (D, E) *N*. *bombycis* coated with BmVg in the oocyte. The arrowhead shows *N*. *bombycis* spores. *N*. *bombycis* spores were stained with FB28 (blue); *N*. *bombycis* in proliferation was labelled by a rabbit anti-*N*. *bombycis* polyclonal antibody conjugated with Alexa Fluor 488 (green); the yellow fluorescence indicates the BmVg labeled by anti-BmVg conjugated with Alexa Fluor 647; nuclei were stained using PI (red). OS, ovariole sheath; FC, follicular cell; O, oocyte; NC, nurse cell.
(TIF)

S5 Fig Determination of pathogen load in pupal fat body and distribution in ovarioles after RNAi. **(**A) Pathogen load in the fat body after RNAi Vg and RNAi GFP treatment. (B) Pathogen load in the fat body after RNAi VgR and RNAi GFP treatment. The pathogen load is represented by copies of *Nb-β-tubulin*. Bars represent the means ± SD of three independent experiments. ns, not significant. (C) The distribution of *N*. *bombycis* in the ovarioles after injection of dsVg, dsVgR and dsGFP. The arrowhead shows the infection site, *N*. *bombycis* spores were stained using FB28 (blue); proliferative *N*. *bombycis* was labelled with antibody conjugated to Alexa Fluor 488 (green); nuclei were stained using PI (red). OS, ovariole sheath; FC, follicular cell; O, oocyte; NC, nurse cell.
(TIF)

S6 Fig The interactions between BmVg domains and spore wall proteins (SWPs). (A) Yeast two-hybrid assay of the interactions between BmVg-VWD and SWP5, SWP7, SWP9, SWP11, SWP16, SWP25, and SWP32. (B) Yeast two-hybrid assay of the interactions between BmVg-DUF1943 and SWP5, SWP7, SWP9, SWP11, SWP16, SWP25, and SWP32. DDO, SD/-Leu/-Trp; QDO, SD/-Ade/-His/-Leu/-Trp. (C) Colocalization of BmVg with SWP12, SWP26 and SWP30 on the spore surface determined by IFA. Spore nuclei were stained using DAPI (blue). BmVg was labeled with anti-BmVg conjugated Alexa Fluor 488 (green). SWP12 (C1), SWP26 (C2), and SWP30 (C3) were marked using antibodies conjugated with Alexa Fluor 594 (red). Bars, 5 μm.
(TIF)

S7 Fig Phylogeny and multiple sequence alignment of lepidopteran vitellogenin. (A) Phylogenetic tree of vitellogenin homologs in lepidoptera insects. A neighbor-joining tree was constructed using MEGA 5 software [73]. Amino acid sequences were obtained from the UniProt database (https://www.uniprot.org). The accession numbers are indicated in brackets. (B) Multiple sequence alignment of vitellogenin sequences among *Bombyx mori*, *Spodoptera litura* and *Helicoverpa armigera*. Alignments were performed using ClustalW [74] and visualized using ESPript [75].
(TIF)

S8 Fig *N*. *bombycis* coated with Vg in the ovarioles of *S*. *litura* and *H*. *armigera*. (A) Verification of polyclonal antibodies specifically recognizing the *S*. *litura* vitellogenin and *H*. *armigera* vitellogenin by western blotting. (B) Spores were isolated from female *S*. *litura* and *H*. *armigera* pupae, and labeled with Vg polyclonal antibody. (C) Colocalization of *N*. *bombycis* and Vg in *S*. *litura* ovarioles. (D) Colocalization of *N*. *bombycis* and Vg in *H*. *armigera* ovarioles. The arrowhead shows the parasite with a Vg signal; *N*. *bombycis* spores were stained using FB28 (blue); proliferative *N*. *bombycis* was labeled using rabbit anti-*N*. *bombycis* polyclonal antibody (Alexa488, green); *S*. *litura* Vg was detected using mouse anti-SlVg polyclonal antibody (Alexa647, yellow); *H*. *armigera* Vg was detected using mouse anti-HaVg polyclonal antibody (Alexa647, yellow); nuclei were stained using PI (red). OS, ovariole sheath; FC, follicular cell; O, oocyte; NC, nurse cell.
(TIF)

S1 Movie Z-stack showing *N*. *bombycis* adhering to the surface of the ovariole aat the previtellogenesis stage on the fifth day of pupation. *N*. *bombycis* spores were stained using FB28 (blue), proliferative *N*. *bombycis* was labeled with antibody conjugated to Alexa Fluor 488 (green), and nuclei were stained using PI (red).
(AVI)

S2 Movie Z-stack showing *N*. *bombycis* adhering to the surface of the ovariole at the vitellogenesis stage on the fifth day of pupation. *N*. *bombycis* spores were stained using FB28 (blue), proliferative *N*. *bombycis* was labeled with antibody conjugated to Alexa Fluor 488 (green), and nuclei were stained using PI (red).
(AVI)

S3 Movie Z-stack showing *N*. *bombycis* infection in follicular cells at the vitellogenesis stage on the fifth day of pupation. *N*. *bombycis* spores were stained using FB28 (blue), proliferative *N*. *bombycis* was labeled with antibody conjugated to Alexa Fluor 488 (green), and nuclei were stained using PI (red).
(AVI)

S4 Movie Z-stack showing *N*. *bombycis* infection in the nurse cells and oocytes at the vitellogenesis stage on the fifth day of pupation. *N*. *bombycis* spores were stained using FB28 (blue), proliferative *N*. *bombycis* was labeled with antibody conjugated to Alexa Fluor 488 (green), and nuclei were stained using PI (red).
(AVI)

S1 Table Primers used in this study. (DOC)

S1 Data Excel Sheets for Graphs. Excel spreadsheet containing in individual tabs, the raw numerical data used to generate the graphs in the following figure panels: Figs 1G, 5B, 5C, 7A, 7C, 7E, 7F, 7G, 9E, 9F and S5.
(XLSX)
