# Supplementary figures and images for: Microsporidian Nosema bombycis hijacks host vitellogenin and restructures ovariole cells for transovarial transmission
Source: PLoS Pathog. 2023 Dec 7;19(12):e1011859. doi: 10.1371/journal.ppat.1011859 (PMC10729982; doi:10.1371/journal.ppat.1011859)

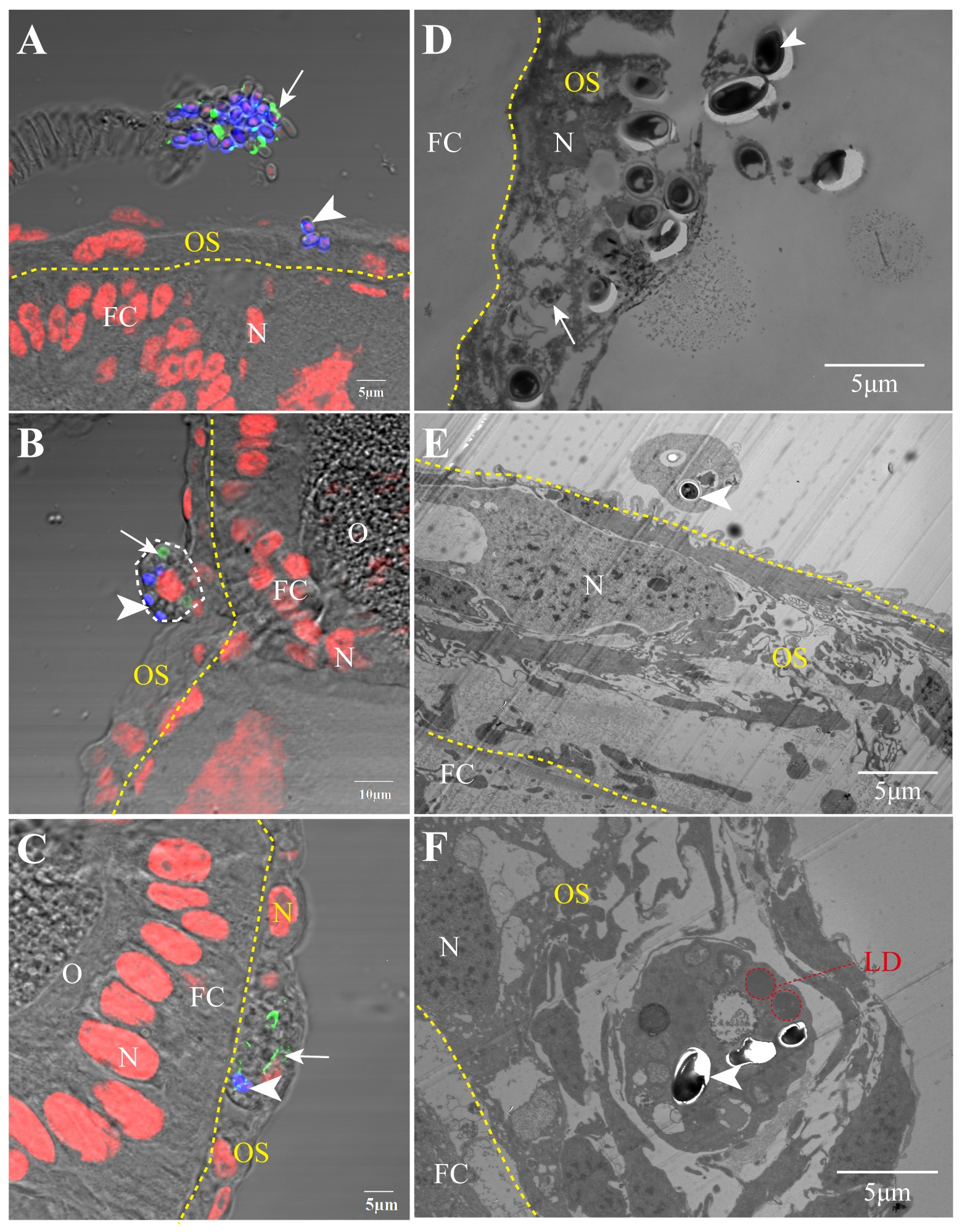

Supplement: S1 Fig — IFA observations show the adhesion of N. bombycis to ovariole sheath (A, B) and proliferation (C). (D-E) TEM demonstrate the parasite proliferation in ovariole sheath. The yellow dashed lines indicate the boundaries of ovariole sheath; white dashed lines indicate the infected hemolymph cells; red dashed lines indicate lipid droplet in the cell. Arrowhead, mature spores; arrow, parasites in proliferation; spores were stained using FB28 (blue); proliferative parasites was labeled with anti-N. bombycis polyclonal antibody and conjugated with Alexa Fluor 488 (green); nuclei were stained using PI (red). N, nucleus; Ov, ovariole; OS, ovariole sheath; LD, lipid droplet; FC, follicular cell; O, oocyte; NC, nurse cell. (TIF) [file ppat.1011859.s001.tif]

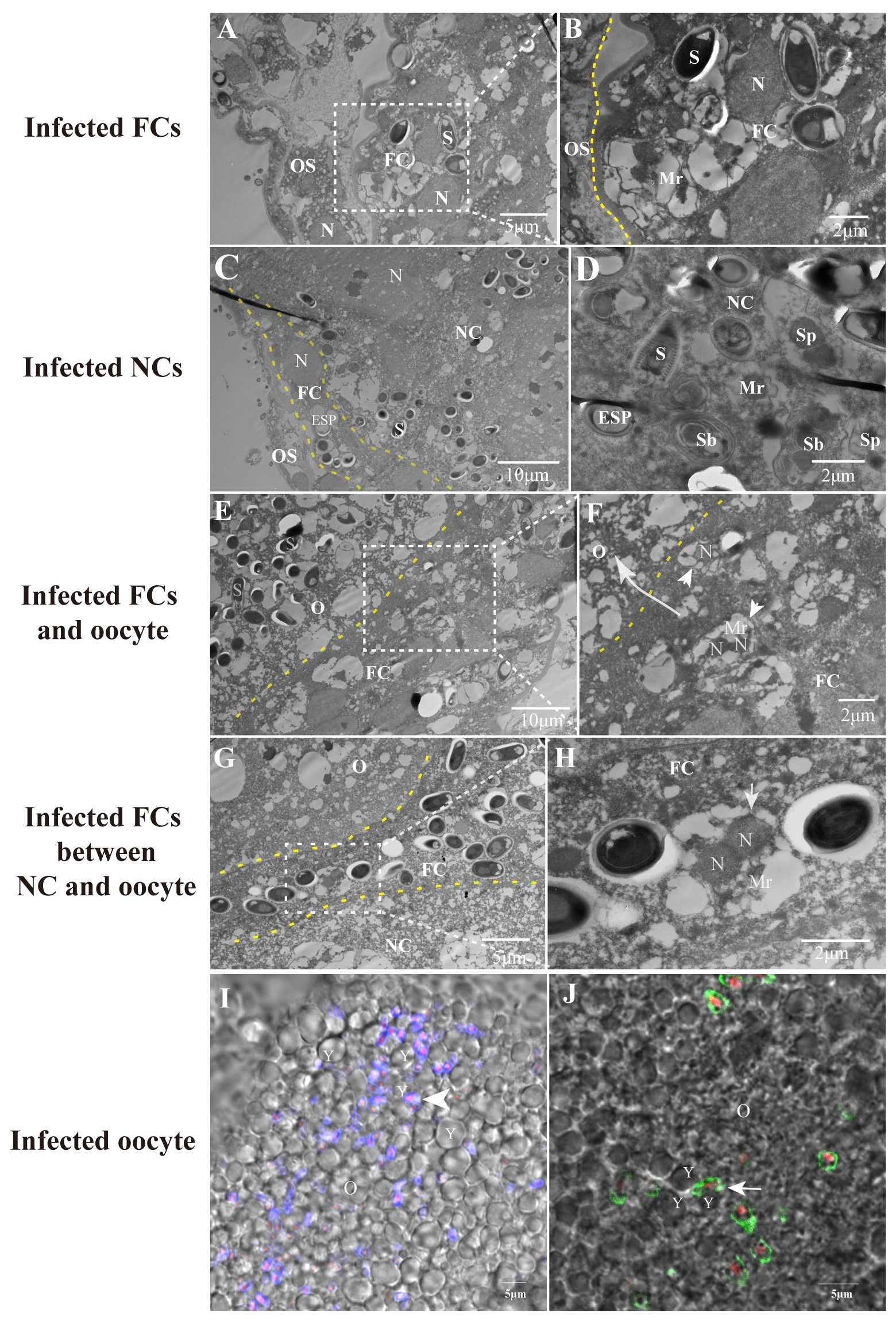

Supplement: S2 Fig — (A, B) TEM demonstrated the infection of FC by N. bombycis. (C, D) TEM analysis of NCs infected by N. bombycis. (E, F) TEM showed the infection of oocyte by N. bombycis via the FC. (G, H) TEM showed the infection of oocyte by N. bombycis from the NC. (I, J) N. bombycis in the oocyte was distributed around the yolk granules. The yellow dashed lines indicate the boundaries of cells. The arrow indicates the N. bombycis; spores were stained using FB28 (blue); N. bombycis in proliferation was labeled with anti-N. bombycis polyclonal antibody conjugated with Alexa Fluor 488 (green); nuclei were stained by PI (red). N, nucleus; S, spore; ESP, empty spore shell; OS, ovariole sheath; FC, follicular cell; O, oocyte; NC, nurse cell; Mr, meront; Sp, sporont; Sb, sporoblast. (TIF) [file ppat.1011859.s002.tif]

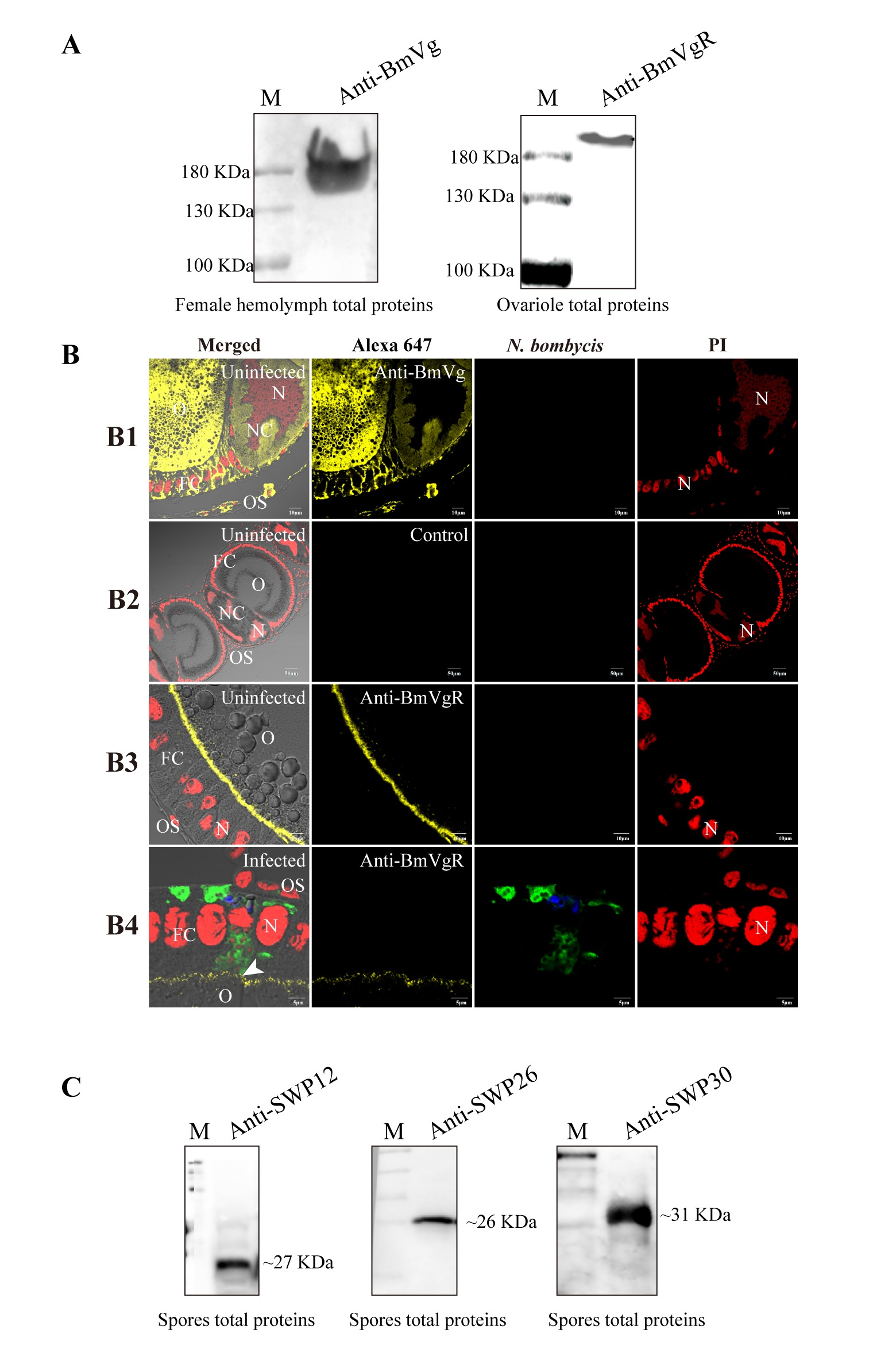

Supplement: S3 Fig — (A) Detection of polyclonal antibodies against BmVg and BmVgR using western blotting. (B) Subcellular localization of BmVg and BmVgR in ovarioles. (B1) Localization of BmVg in uninfected ovarioles. (B2) Negative serum was used as the control. (B3) Localization of VgR in uninfected ovarioles. (B4) Localization of N. bombycis spores and BmVgR in oocytes. (C) Detection of polyclonal antibodies that recognize the N. bombycis spore wall protein 12, spore wall protein 26, and spore wall protein 30 using western blotting. The arrowhead shows N. bombycis; N. bombycis spores were stained using FB28 (blue); proliferative N. bombycis were labelled with rabbit polyclonal antibody against N. bombycis (Alexa488, green); BmVg was detected using a mouse anti-BmVg polyclonal antibody (Alexa647, yellow); nuclei were stained using PI (red). OS, ovariole sheath; FC, follicular cell; O, oocyte; NC, nurse cell. (TIF) [file ppat.1011859.s003.tif]

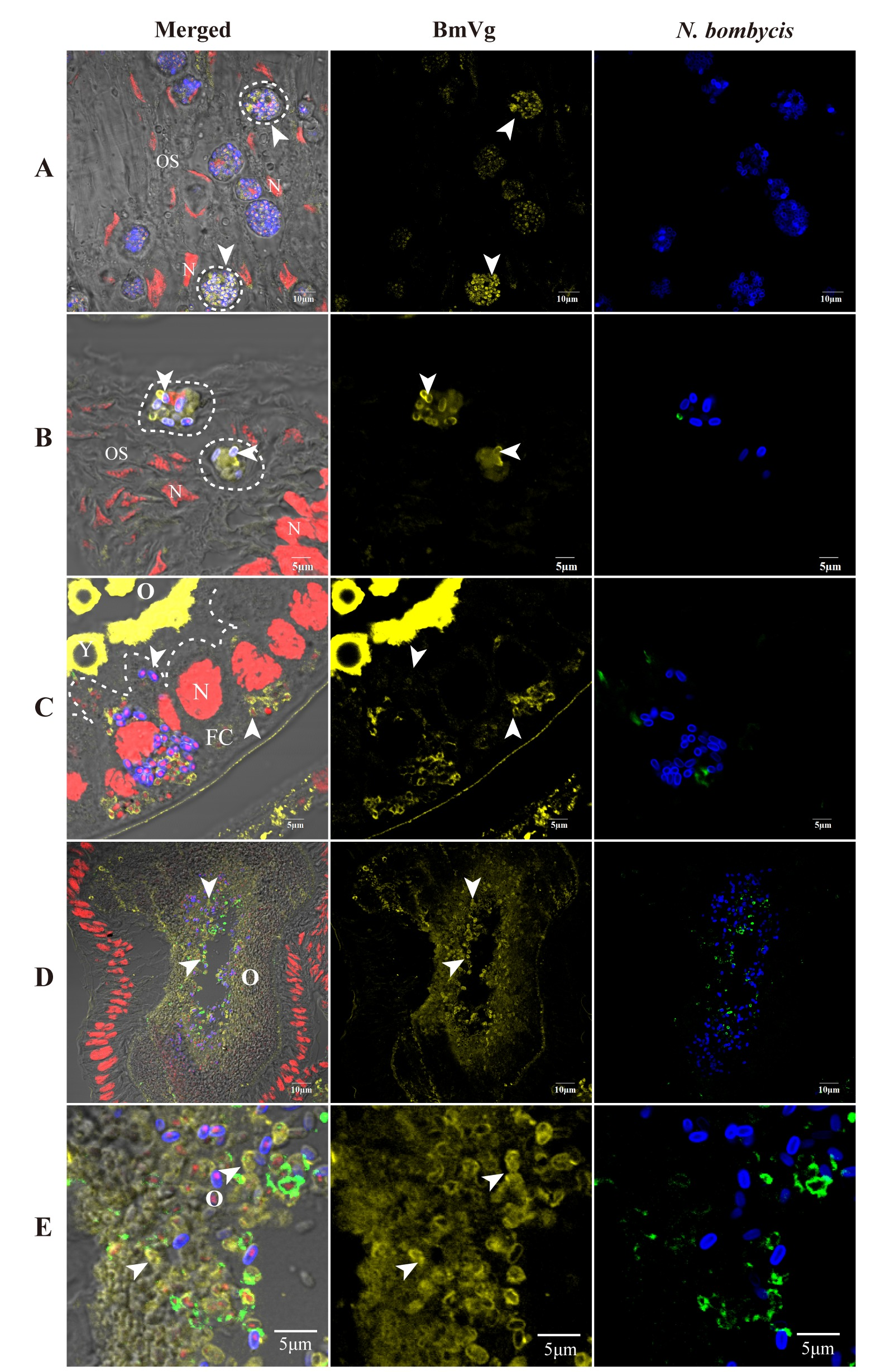

Supplement: S4 Fig — (A, B) N. bombycis coated with BmVg in the ovariole sheath cells, the white dashed lines indicate the infected hemolymph cells. (C) N. bombycis coated with BmVg during infecting the oocyte from FCs, the white dashed lines indicate the boundaries of FCs. (D, E) N. bombycis coated with BmVg in the oocyte. The arrowhead shows N. bombycis spores. N. bombycis spores were stained with FB28 (blue); N. bombycis in proliferation was labelled by a rabbit anti-N. bombycis polyclonal antibody conjugated with Alexa Fluor 488 (green); the yellow fluorescence indicates the BmVg labeled by anti-BmVg conjugated with Alexa Fluor 647; nuclei were stained using PI (red). OS, ovariole sheath; FC, follicular cell; O, oocyte; NC, nurse cell. (TIF) [file ppat.1011859.s004.tif]

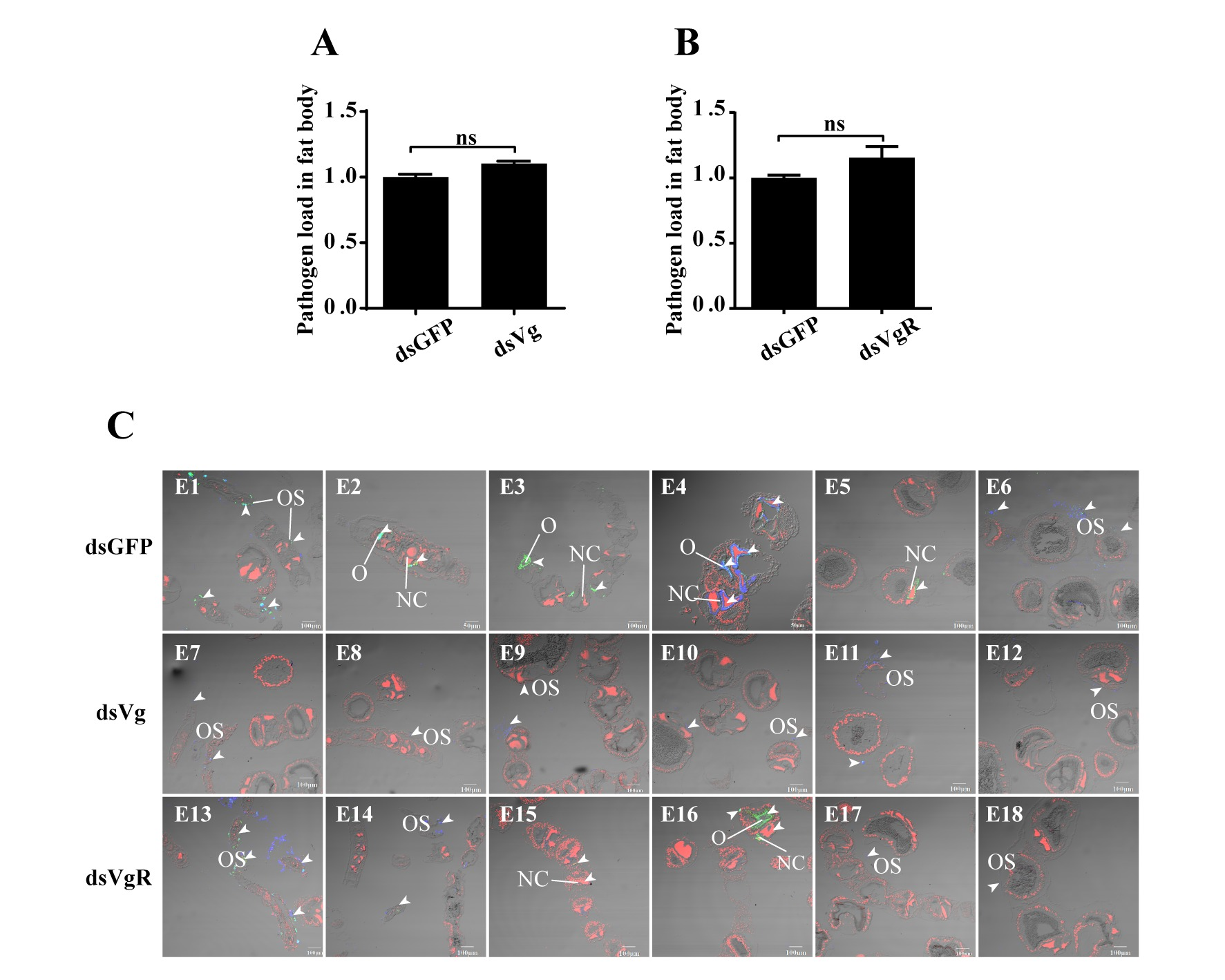

Supplement: S5 Fig — (A) Pathogen load in the fat body after RNAi Vg and RNAi GFP treatment. (B) Pathogen load in the fat body after RNAi VgR and RNAi GFP treatment. The pathogen load is represented by copies of Nb-β-tubulin. Bars represent the means ± SD of three independent experiments. ns, not significant. (C) The distribution of N. bombycis in the ovarioles after injection of dsVg, dsVgR and dsGFP. The arrowhead shows the infection site, N. bombycis spores were stained using FB28 (blue); proliferative N. bombycis was labelled with antibody conjugated to Alexa Fluor 488 (green); nuclei were stained using PI (red). OS, ovariole sheath; FC, follicular cell; O, oocyte; NC, nurse cell. (TIF) [file ppat.1011859.s005.tif]

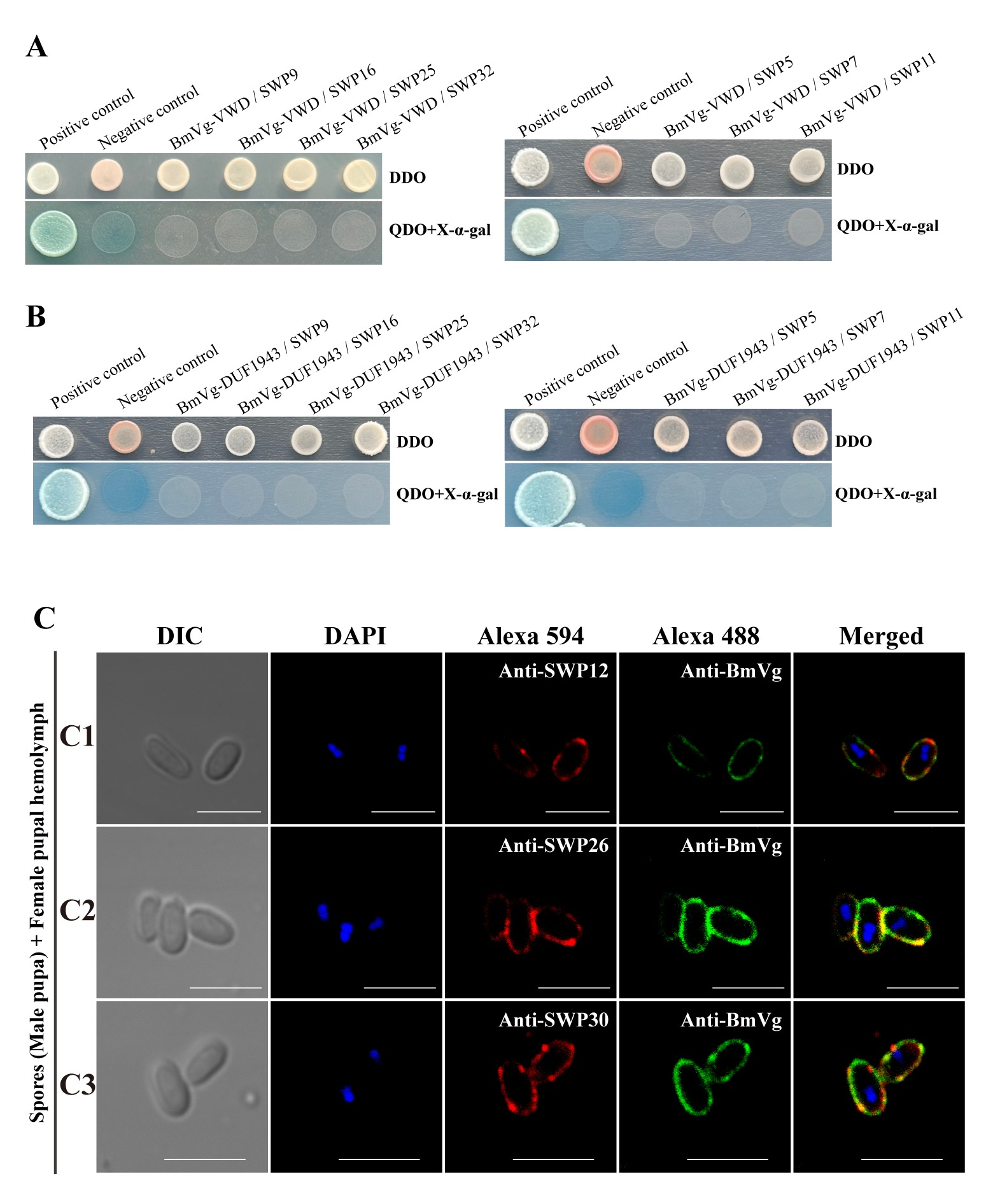

Supplement: S6 Fig — (A) Yeast two-hybrid assay of the interactions between BmVg-VWD and SWP5, SWP7, SWP9, SWP11, SWP16, SWP25, and SWP32. (B) Yeast two-hybrid assay of the interactions between BmVg-DUF1943 and SWP5, SWP7, SWP9, SWP11, SWP16, SWP25, and SWP32. DDO, SD/-Leu/-Trp; QDO, SD/-Ade/-His/-Leu/-Trp. (C) Colocalization of BmVg with SWP12, SWP26 and SWP30 on the spore surface determined by IFA. Spore nuclei were stained using DAPI (blue). BmVg was labeled with anti-BmVg conjugated Alexa Fluor 488 (green). SWP12 (C1), SWP26 (C2), and SWP30 (C3) were marked using antibodies conjugated with Alexa Fluor 594 (red). Bars, 5 μm. (TIF) [file ppat.1011859.s006.tif]

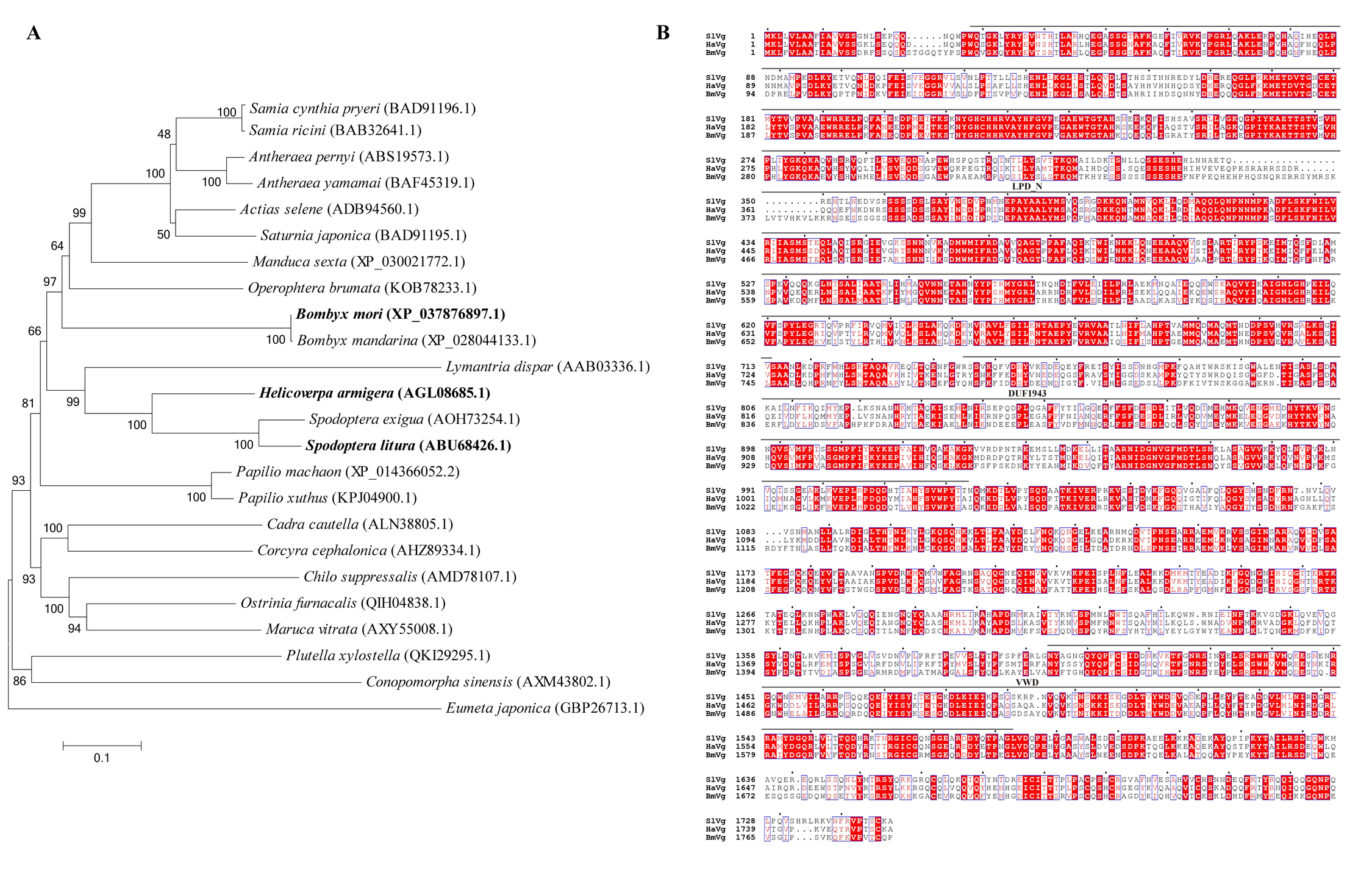

Supplement: S7 Fig — (A) Phylogenetic tree of vitellogenin homologs in lepidoptera insects. A neighbor-joining tree was constructed using MEGA 5 software [73]. Amino acid sequences were obtained from the UniProt database (https://www.uniprot.org). The accession numbers are indicated in brackets. (B) Multiple sequence alignment of vitellogenin sequences among Bombyx mori, Spodoptera litura and Helicoverpa armigera. Alignments were performed using ClustalW [74] and visualized using ESPript [75]. (TIF) [file ppat.1011859.s007.tif]

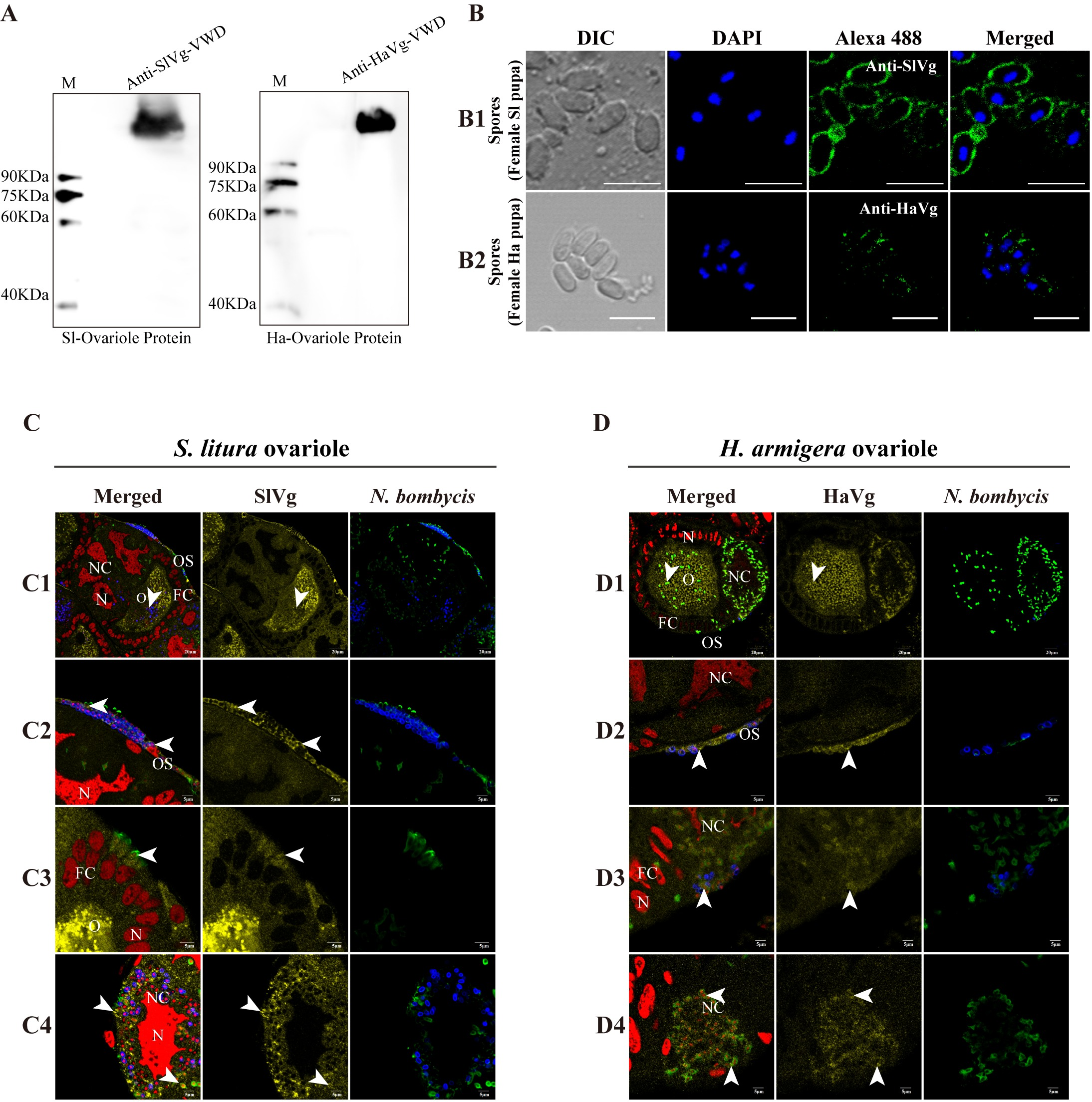

Supplement: S8 Fig — (A) Verification of polyclonal antibodies specifically recognizing the S. litura vitellogenin and H. armigera vitellogenin by western blotting. (B) Spores were isolated from female S. litura and H. armigera pupae, and labeled with Vg polyclonal antibody. (C) Colocalization of N. bombycis and Vg in S. litura ovarioles. (D) Colocalization of N. bombycis and Vg in H. armigera ovarioles. The arrowhead shows the parasite with a Vg signal; N. bombycis spores were stained using FB28 (blue); proliferative N. bombycis was labeled using rabbit anti-N. bombycis polyclonal antibody (Alexa488, green); S. litura Vg was detected using mouse anti-SlVg polyclonal antibody (Alexa647, yellow); H. armigera Vg was detected using mouse anti-HaVg polyclonal antibody (Alexa647, yellow); nuclei were stained using PI (red). OS, ovariole sheath; FC, follicular cell; O, oocyte; NC, nurse cell. (TIF) [file ppat.1011859.s008.tif]
