# Supplementary material for: Microsporidian Nosema bombycis hijacks host vitellogenin and restructures ovariole cells for transovarial transmission
Source: PLoS Pathog. 2023 Dec 7;19(12):e1011859. doi: 10.1371/journal.ppat.1011859 (PMC10729982; doi:10.1371/journal.ppat.1011859)
Supplement: S1 Table — (DOC) [file ppat.1011859.s013.doc]

S1 Table Primers used in this study.

| Purpose | Construct | Primer sequences | | |
| --- | --- | --- | --- | --- |
|  |  | Forward |  | Reverse |
| For construction of expression vector | pET30-BmVg | 5'-GCTTGTCGACGGAGCTCGAATTCTCTATCGATGGCACAAGG-3' |  | 5'-CGACAAGCTTGCGGCCGCACTCGAGCAAACCTTCTGGAGTGAGAT -3' |
|  | pET30-BmVgR | 5'-GCTTGTCGACGGAGCTCGAATTCAGTGCCTGGGCGAGGATGTG -3' |  | 5'-CGACAAGCTTGCGGCCGCACTCGAGGATGCATCTGCCGTTCTTGTTTC-3' |
|  | pCold-TF-LPDN-1 | 5'-TCGTCGACGGATCCGAATTCATGTATCCATCACCTTGGCA-3' |  | 5'-CTTATCGTCGTCATCCTTGTAATCAGACGAGCTTTCGCTGTTG-3' |
|  | pCold-TF-LPDN-2 | 5'-TCGTCGACGGATCCGAATTCGGTTCTTCAAGTTCGAGTGC-3' |  | 5'-CTTATCGTCGTCATCCTTGTAATCTTATAATTCAGCAGCTGATA  -3 |
|  | pCold-TF-DUF1943 | 5'-CTCGGTACCCTCGAGGGATCCATGCAGCATTCATTTAAGTTTATT-3' |  | 5'-CTTATCGTCGTCATCCTTGTAATCGTACGGCCAAACACTATAGTGAA-3' |
|  | pET32-VWD | 5'-AAGGCCATGGCTGATATCGGATCCATGAATTACTTCACTGGCCACCA-3' |  | 5'-CGTAGAATCGAGACCGAGGAGAGGGTTAGGGATAGGCTTACCCAAACCTTCTGGAGTGAGAT-3' |
| Yeast two hybrid assay | pGADT7-VWD | 5'-CGACGTACCAGATTACGCTCATATGATGGTCTCATTCCAAGACATG-3' |  | 5'-GCAGCTCGAGCTCGATGGATCCTTAAGGCTTGTCGACCAAACCTTC-3' |
|  | pGADT7-DUF1943 | 5'-CGACGTACCAGATTACGCTCATATGATGCAGCATTCATTTAAGTTTATT-3' |  | 5'- GCAGCTCGAGCTCGATGGATCCATCCTTCTTTTGGCTGGCAC-3' |
|  | pGBKT7-SWP12 | 5'-ATCTCAGAGGAGGACCTGCATATGATGAAAGATTTTAAAAAG-3' |  | 5'- GGCCGCTGCAGGTCGACGGATCCCTTAGTCCTCTCTAATGC-3' |
|  | pGBKT7-SWP26 | 5'-GGCCGCTGCAGGTCGACGGATCCCTTAGTCCTCTCTAATGC-3' |  | 5'- GGCCGCTGCAGGTCGACGGATCCATCATCAATTCCAGAAAT-3' |
|  | pGBKT7-SWP30 | 5'-ATCTCAGAGGAGGACCTGCATATGATGAATATTTTACTTGCT-3' |  | 5'- GGCCGCTGCAGGTCGACGGATCCGAAAGGAATGGTATTGTC-3' |
| dsRNA assay | T7-GFP | 5'-TAATACGACTCACTATAGGGAGATGCTTCAGCCGCTACCC-3' |  | 5'-TAATACGACTCACTATAGGGAGATCCAGCAGGACCATGTGAT  -3' |
|  | T7-Vg | 5'-TAATACGACTCACTATAGGGAGAACTAATAAAGTTCAGAGTCG - 3' |  | 5'-TAATACGACTCACTATAGGGAGACAGCAGAAACTAGTTTCACCA -3' |
|  | T7-VgR | 5'-TAATACGACTCACTATAGGGAGAAGATGGACACTTCGGTATTT -3' |  | 5'-TAATACGACTCACTATAGGGAGAGTTGACGGTGACGATGTG - 3' |
| RT-qPCR assay | q-Vg | 5'-AGTCACGACGAATACCAAGAAGAT-3' |  | 5'-TACGATAGTCCTGTGTGAAAACGA- 3' |
|  | q-VgR | 5'-AGTGCCTGGGCGAGGATGT-3' |  | 5'-ACTGAGCGTCTGGCTTGTGA- 3' |
|  | q-Nb-β-tubulin | 5'-AGAACCAGGAACAATGGACG-3' |  | 5'- AGCCCAATTATTACCAGCACC-3' |
|  | q-RPL3 | 5'-CGGTGTTGTTGGATACATTGAG-3' |  | 5'-GCTCATCCTGCCATTTCTTACT-3' |
| Pull-down assay | pMAL-c5X-SWP12 | 5'-CATATGTCCATGGGCGGCCGCATGAAAGATT  TTAAAAAGAAA-3' |  | 5'-CTTATCGTCGTCATCCTTGTAATCCTTAGTCCTCT  CTAATGC-3' |
|  | pMAL-c5X-SWP26 | 5'-GGATTTCACATATGTCCATGGGCATGAATATTA  TCATCTTCAGC-3' |  | 5'-CTTATCGTCGTCATCCTTGTAATCATCATCAATTC  CAGAAAT-3' |
|  | pMAL-c5X-SWP30 | 5'-GGATTTCACATATGTCCATGGGCATGAATATTTT  ACTTGCTACA-3' |  | 5'-CTTATCGTCGTCATCCTTGTAATCGAAAGGAATG  GTATTGTC-3' |
